# Minimal Clinically Important Difference of Scales Reported in Stroke Trials: A Review

**DOI:** 10.3390/brainsci14010080

**Published:** 2024-01-13

**Authors:** Biswamohan Mishra, Pachipala Sudheer, Ayush Agarwal, Nilima Nilima, Madakasira Vasantha Padma Srivastava, Venugopalan Y. Vishnu

**Affiliations:** 1 Department of Neurology, All India Institute of Medical Sciences, New Delhi 110029, India; biswamohan26@gmail.com (B.M.); mail2sudhirpamc@gmail.com (P.S.); ayushthetaurian@gmail.com (A.A.); vasanthapadma123@gmail.com (M.V.P.S.); 2 Department of Biostatics, All India Institute of Medical Sciences, New Delhi 110029, India; nilima3012@gmail.com

**Keywords:** stroke, minimal clinically important difference, minimal clinically important change, clinical relevance, patient-reported outcome measures (PROMs), MCID

## Abstract

There is a growing awareness of the significance of using minimum clinically important differences (MCIDs) in stroke research. An MCID is the smallest change in an outcome measure that is considered clinically meaningful. This review is the first to provide a comprehensive summary of various scales and patient-reported outcome measures (PROMs) used in stroke research and their MCID values reported in the literature, including a concise overview of the concept of and methods for determining MCIDs in stroke research. Despite the controversies and limitations surrounding the estimation of MCIDs, their importance in modern clinical trials cannot be overstated. Anchor-based and distribution-based methods are recommended for estimating MCIDs, with patient self-evaluation being a crucial component in capturing the patient’s perspective on their health. A combination of methods can provide a more comprehensive understanding of the clinical relevance of treatment effects, and incorporating the patient’s perspective can enhance the care of stroke patients.

## 1. Introduction

The origin of evidence-based medicine (EBM) dates back to the 1970s. This paradigm emphasizes a methodical evaluation of the evidence for use in health care decision-making, along with the knowledge of decision-makers and the expectations and values of patients. There is a growing awareness of correlating statistically significant results with clinical relevance in clinical trials to avoid the misinterpretation of study findings and prevent patients from being exposed to unnecessary therapies [1,2]. The concept of “clinically important difference”, which has been developed as a way to overcome the drawbacks of a “statistically significant difference” and which represents a change that the patient feels, is noteworthy. The minimum clinically important difference (MCID) is the threshold value for such a change [3], first described by Jaeschke and colleagues in 1989 [4]. There has been a shift towards considering clinical relevance rather than just statistical significance in interpreting results from clinical trials [5].

Multiple rating scales, such as the Modified Rankin Scale (mRS) and the Barthel Index (BI), are commonly used as outcome measures in both daily neurological practice and clinical trials, including stroke trials [6]. Understanding the MCID in stroke trials is essential for several reasons.

Firstly, determining the MCID is crucial for sample size calculations in stroke trials. An accurate estimation of the MCID can help to ensure that the trial is adequately powered to detect a clinically significant treatment effect. If the MCID is small, smaller sample sizes may be sufficient to detect meaningful differences between treatment groups, which can reduce the cost and duration of the trial [7].

Secondly, understanding the MCID can help to interpret the clinical relevance of treatment effects. The MCID can help to determine whether a treatment effect is large enough to be clinically meaningful for patients [8].

Thirdly, the MCID can guide the development of new outcome measures that are more sensitive to clinically meaningful changes. Outcome measures that have a smaller MCID are more likely to detect smaller but clinically meaningful changes in patient outcomes, which can improve the sensitivity of stroke trials and enhance their ability to detect treatment effects.

Lastly, understanding the MCID can aid in the selection of appropriate endpoints in stroke trials. In some cases, the endpoints used in clinical trials may not align with patient-centered outcomes or may not have a meaningful MCID. Understanding the MCID can help to identify appropriate endpoints that are more relevant and meaningful to patients [8].

Therefore, the MCID is a crucial concept in stroke trials that can help to ensure that trials are adequately powered to detect meaningful treatment effects, aid in the interpretation of treatment outcomes, guide the development of new outcome measures, and aid in the selection of appropriate endpoints. Incorporating the MCID into stroke trials can improve the quality of stroke research and enhance the care of stroke patients.

This review aims to familiarize clinicians with the definition and methods for the estimation of the MCID, different patient-reported outcome measures (PROMs) in stroke, and their use in the calculation of the MCID for stroke-related scales.

## 2. Literature Search

References for this review were identified by searching PubMed, Google Scholar, Embase, and MEDLINE till Jan 2023 as well as searching references from relevant articles. The search terms used were (“MCID” OR “MID” OR “minimal clinically important difference” OR “minimal important difference” OR “minimal clinically important change” OR “clinically important change” OR “minimal clinical important difference” OR “clinical important difference” OR “meaningful change”) AND (“stroke”). The search was restricted to English-language articles. The final reference list was generated on the basis of relevance to the topics covered in this review.

## 3. Statistical Significance of MCIDs in Clinical Trials

### 3.1. Trials Evaluating Superiority

The MCID is an important consideration in sample size determination for clinical trials because it helps to ensure that the trial is large enough to detect a difference in outcomes that is meaningful to patients [9].

**Figure 1:**
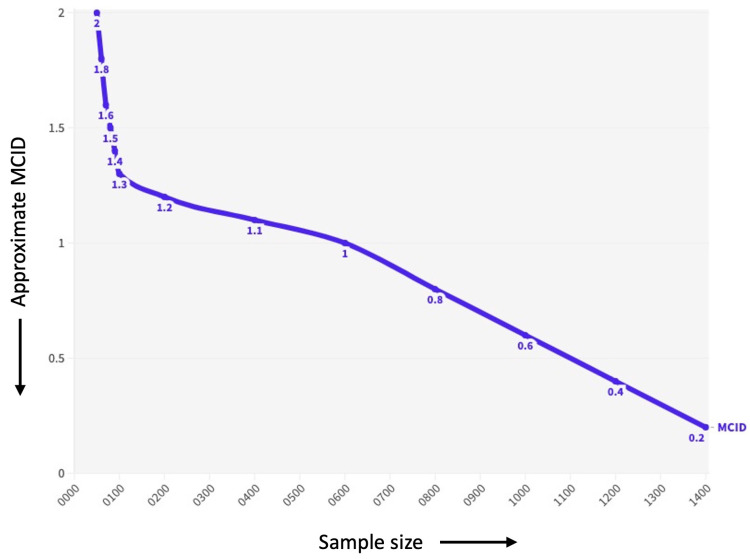
Figure shows the relationship between hypothetical MCID (Minimal Clinically Important Difference) values and sample size. The *y*-axis shows the MCID in points and the *x*-axis shows the required sample size in patients.

In the context of power to detect a treatment effect (superiority), a larger MCID will require a larger sample size to detect a statistically significant difference between treatments. This is because the MCID represents the smallest difference that is likely to be important to patients, so the trial needs to be large enough to have a high probability of detecting a difference of that size (Figure 1).

For example, let us say a clinical trial is being planned to compare a new drug to a placebo for the treatment of stroke. It is hypothesized that the new drug is effective, but to make sure of that, a large enough sample size is needed to detect a difference in stroke scores that is likely to be important to patients. An MCID of 10 points on a 100-point ABC scale is decided as the MCID for the stroke score. This means that the researchers would be 90% confident that they can detect a difference in pain scores of at least 10 points between the new drug and placebo groups if there is truly a difference in efficacy.

Using a standard sample size calculator, it can be estimated that a sample size of 120 patients is needed in each treatment group to achieve a power of 90% to detect a difference of 10 points on the ABC scale. If we used a smaller MCID, such as five points, we would need a larger sample size of 200 patients in each group.

### 3.2. Trials Evaluating Equivalence and Non-Inferiority

In the context of power to show similarity (equivalence and non-inferiority trials), a smaller MCID will require a larger sample size to show that two treatments are similar. This is because the MCID represents the smallest difference that is likely to be clinically meaningful, so the trial needs to be large enough to have a high probability of showing that the two treatments do not differ by more than that amount.

For example, let us say a clinical trial is being planned to compare a new drug to an existing drug for the treatment of hypertension. We want to show that the new drug is not inferior to the existing drug in terms of blood pressure control. We decide to use an MCID of 2 mmHg for systolic blood pressure. This means that we want to be 90% confident that we will not detect a difference in systolic blood pressure of more than 2 mmHg between the new drug and existing drug groups, if the two drugs are truly equivalent.

Using a standard sample size calculator, we can estimate that we need a sample size of 400 patients in each treatment group to achieve a power of 90% to show the non-inferiority of the new drug to the existing drug by 2 mmHg. If we used a larger MCID, such as 5 mmHg, we would need a smaller sample size of 200 patients in each group (Figure 1).

## 4. Definitions and Approaches to Estimate MCID

A patient-reported outcome measure (PROM) is defined as “any report coming directly from patients about how they function or feel in relation to a health condition and its therapy” [10]. The ability of an instrument to measure significant change over time is called responsiveness of the instrument. These properties in turn affect the design of clinical trials and their sample size, and also have a bearing on the interpretation of results [7].

The term MCID was first defined by Jaeschke et al. as “the smallest difference in score in the domain of interest which patients perceive as beneficial and which would mandate, in the absence of troublesome side effects and excessive cost, a change in the patient’s management” [4].

There are three main approaches to determining the MCID (minimum clinically important difference): anchor-based methods, distribution-based methods, and the Delphi method. All of these approaches measure a change in outcome, but the specific approach used will determine the type of change that is measured.

**Figure 2:**
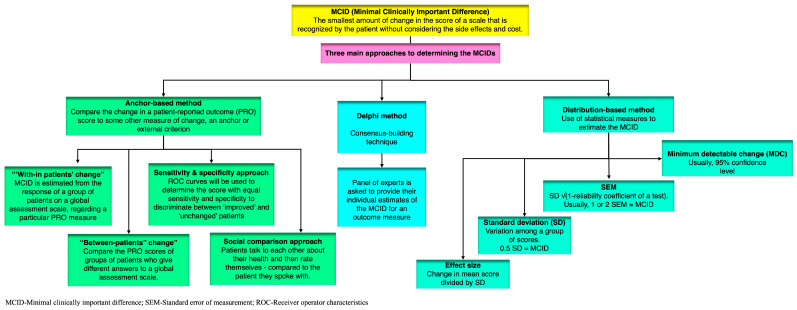
Figure illustrates the various methods and approaches used in the estimation of the Minimal Clinically Important Difference (MCID) in patient-reported outcome measures (PROMs) and other clinical assessments.

The choice of approach depends on the specific situation and the type of outcome measure being used. In general, anchor-based methods are considered to be more reliable, but they can be more difficult to implement. Distribution-based methods are less reliable, but they are easier to implement. Figure 2 illustrates the varied methods and approaches used in the estimation of the MCID in patient-reported outcome measures (PROMs) and other clinical assessments.

### 4.1. Anchor-Based Approach

Anchor-based approaches compare the change in a patient-reported outcome (PRO) score to some other measure of change, called an anchor or external criterion. The anchor can be either objective or subjective.

a. Objective anchors are based on physical measurements, such as the amount of pain medication a patient takes or the number of steps they can walk. These anchors are more reliable than subjective anchors, but they are not always available.
b. Subjective anchors are based on the patient’s own assessment of their health, such as how much better or worse they feel. These anchors are less reliable than objective anchors, but they are more commonly used because they are easier to obtain.

Some examples of objective anchors include:

- The amount of pain medication a patient takes;
- The number of steps a patient can walk;
- The patient’s functional status (e.g., their ability to bathe, dress, or walk);
- The patient’s quality of life.

Some examples of subjective anchors include:

- The patient’s global assessment of their health (e.g., “better”, “worse”, or “unchanged”) (e.g., patient global impression of change (PGIC) or global rating of change (GROC));
- The patient’s rating of their pain on a scale of 0 to 10;
- The patient’s rating of their overall health on a scale of 1 to 10;
- The clinician’s rating of the patient’s overall health on a scale of 1 to 10 (e.g., clinician global impression of change (CGIC)).

Interpreting patient-reported outcome (PRO) scores through anchor-based approaches is an evolving practice. The choice of anchor plays a crucial role in determining what magnitude of change in the PRO score is considered meaningful. For example, if the anchor is the amount of pain medication a patient takes, then a change of 10% in the PRO score might be considered meaningful. However, if the anchor is the patient’s rating of their overall health, then a change of 20% in the PRO score might be considered meaningful.

The strength of the association between the PRO score and the external criterion also affects how PRO scores are interpreted. A strong correlation suggests that changes in the PRO score are likely to be accompanied by changes in the external criterion. Research suggests that a correlation coefficient of at least 0.3 is necessary to make reliable inferences [11].

Objective external criteria are seldom used in studies, with most studies relying on patients’ subjective assessments or global assessment scales, leading to ongoing debates regarding their validity and reliability. The reliance on subjective assessments arises from the lack of satisfactory objective assessments, prompting the use of PROs in the first place [12].

Efforts have been made to validate patients’ subjective assessments by combining them with clinicians’ evaluations or considering physical therapists’ reports, yet the choice of external criterion remains critical [12,13,14]. Regardless of the chosen criterion, a well-established association between the criterion and the PRO measurement is essential for drawing meaningful conclusions [15]. Despite these efforts, anchor-based methods are vulnerable to recall bias, where recent events are better remembered than those in the distant past, and to the influence of the patient’s current health status on reported changes, underscoring the complexity of accurately capturing treatment effects through these methods [16]. Nevertheless, global assessment scales have demonstrated high sensitivity to change, both positive and negative [17].

While employing an external criterion is a shared feature in all anchor-based methods, several distinctions persist among these approaches. Four distinct variations can be discerned within anchor-based methodologies.

#### 4.1.1. “With-in Patients” Score Change

In this method, the MCID is estimated from the response of a group of patients on a global assessment scale regarding a particular PRO measure. Typical anchor-based studies have a Likert-type scale, like a 15-point global scale (−7 = ‘‘much worse’’ to 0 = ‘‘no change’’ to +7 = ‘‘much better’’), to record patient-reported changes. In early studies, the MCID was calculated as the average change for patients who reported small changes, meaning they rated themselves as slightly better (scores of 1, 2, or 3) [4]. Later, a score of 1 was treated as equivalent to 0, and only patients with scores of 2 or 3 were used to calculate MCID [18]. Similar techniques have been used with different scales; for example, some studies use a six-point scale, and the MCID is determined based on the mean change in scores of patients who reported being “much improved” [19].

#### 4.1.2. “Between-Patients” Score Change

Another way to define the MCID is to compare the PRO scores of groups of patients who give different answers to a global assessment scale.

For example, in a cross-sectional study, the PRO scores of patients who say they are “not at all impaired” could be compared to the PRO scores of patients who say they are “very mildly impaired.” The MCID would then be defined as the difference in PRO scores between these two groups.

In a longitudinal study, we could compare the PRO scores of patients who say they have gotten “better” to the PRO scores of patients who say they have stayed “unchanged”. The MCID would then be defined as the difference in PRO scores between these two groups [17,20].

#### 4.1.3. Sensitivity- and Specificity-Based Approach

This approach aims to find an MCID that best distinguishes between groups of patients. Sensitivity is how well a test identifies patients with a condition, while specificity is how well it identifies those without the condition. In this context, sensitivity means the proportion of patients who report an improvement in an external criterion and have PRO scores above the MCID value. Specificity means the proportion of patients who do not report improvement and have PRO scores below the MCID value. A sensitivity of 1 means all true positives are identified, while a specificity of 1 means all true negatives are identified [17]. There is no agreed-upon ideal sensitivity or specificity level for the MCID, but researchers often aim for balance.

Receiver operating characteristic (ROC) curves are frequently used to identify the PRO score that best distinguishes between “improved” and “unchanged” patients [12,14,19]. The area under the ROC curve reflects how well scores discriminate between these groups. An area of 0.7 to 0.8 is considered good, and 0.8 to 0.9 is excellent for accuracy, but the choice of patient groups for ROC analysis can still be somewhat arbitrary [21].

#### 4.1.4. Social Comparison Approach

In a less commonly used approach, patients talk to each other about their health and then rate themselves as “a little better,” “a little worse”, or “about the same” compared to the patient they spoke with. The MCID is the score difference between those who rate themselves as “a little better” or “a little worse” and those who say they are “about the same” as the other patient [22].

### 4.2. Distribution-Based Approaches

Distribution-based approaches use statistical measures to determine the MCID. These include those mentioned in the following sections.

#### 4.2.1. SEM (Standard Error of Measurement)

The SEM represents a variation in scores due to measurement unreliability. If a change is smaller than the SEM, it is likely due to measurement error rather than a real change. At the least, 1 SEM may be used as the yardstick of true change for individual or group change scores, but there is no consensus on a general value [23,24].

#### 4.2.2. MDC (Minimum Detectable Change)

The MDC is the smallest change that can be considered real, rather than a measurement error, with a certain level of confidence (usually 95%) [25]. A valid MCID should be at least as large as the observed MDC [17].

#### 4.2.3. SD (Standard Deviation)

Some studies have found that 0.5 SD corresponds to the MCID. This is because 0.5 SD represents the limit of human discriminative capacity and is equivalent to 1 SEM with a reliability of 0.75 [26].

#### 4.2.4. Effect Size

Effect size measures change by comparing post-treatment scores to baseline scores, standardized by the baseline score’s standard deviation. An effect size of 0.2 is small, 0.5 is moderate, and 0.8 is large [25]. The MCID corresponds to the change score associated with a small effect size (0.2), calculated by multiplying the baseline score’s SD by 0.2 [27].

Distribution-based methods solely rely on statistical approaches without incorporating clinical questionnaires and are tailored to the specific characteristics of the patient cohort. While they can capture changes beyond random variation, these methods lack agreed-upon benchmarks for establishing clinically significant improvement. Furthermore, they do not consider the patient’s perspective of what constitutes a clinically important change, which differs significantly from statistical significance [3,28].

### 4.3. The Delphi Method

A lesser-used approach to estimate the MCID is the Delphi method. The Delphi method is a consensus-building technique that can be used to estimate the minimally important change (MCID) for an outcome measure. In the Delphi method, a panel of experts is asked to provide their individual estimates of the MCID. The experts are then given feedback on the range of estimates provided by the other experts. This process is repeated until a consensus is reached on the MCID.

The Delphi method is particularly well-suited for estimating the MCID for technical efficacy outcomes, such as reperfusion after stroke. These outcomes are often difficult for patients to assess, and so expert opinion is needed to determine the smallest change that is likely to be clinically meaningful [29].

However, it can be a time-consuming process, as it may take several rounds of feedback to reach a consensus on the MCID. Also, it is subject to the biases of the panel of experts. If the panel is not representative of the population of experts, then the MCID may not be generalizable to the wider population [30].

### 4.4. Case Scenario: Hypothetical Illustration

Consider the example of a commonly used outcome measure in stroke, i.e., the Modified Rankin Scale (mRS), a six-point scale that is used to assess disability after stroke.

Method: Distribution-based method; estimated MCID: one-point change.

A one-point change in mRS score is considered to be a minimally important change because it is associated with a noticeable difference in the patient’s level of disability. For example, a patient who moves from a score of 3 (moderately disabled) to a score of 2 (slightly disabled) is likely to experience a significant improvement in their ability to function independently.

Method: Anchor-based method.

MCID: Change in mRS score that is associated with a minimally important change in a patient-reported outcome measure (PROM), such as the Stroke Impact Scale (SIS).

For example, a study might find that a one-point change in mRS score is associated with a ten-point change on the SIS. This would suggest that a one-point change in mRS score is a minimally important change because it is associated with a noticeable difference in the patient’s self-reported quality of life.

Method: Expert consensus/Delphi method.

MCID: Change in mRS score that is identified by a panel of experts as being the smallest change that they would consider to be clinically important.

For example, a panel of stroke experts might be asked to estimate the smallest change in mRS score that they would consider to be a minimally important change. The experts might agree that a one-point change in mRS score is a minimally important change because it is associated with a noticeable difference in the patient’s overall neurological status.

### 4.5. Choice of Method Depending on Outcome Measure

Various approaches for establishing the minimal clinically important difference (MCID) might be more appropriate depending on the nature of the outcomes. For example, anchor-based methods may be more appropriate for patient-reported outcomes (PROMs) because they compare the change in a scale-based outcome measure with that of a patient-reported outcome or other external criterion. On the other hand, distribution-based methods may be more appropriate for objective measures because they compare the difference in a scale-based outcome measure to a prespecified threshold value of its uncertainty, which facilitates MCID derivation when direct patient or clinician input is not readily accessible [31].

### 4.6. Limitations of MCID

The estimation of MCID has its own limitations. Three general limitations in the accurate determination of an MCID have been identified: the multiplicity of MCID determinations, the loss of the patient’s perspective, and the relationship between the pretreatment baseline and post-treatment score change.

#### 4.6.1. Multiplicity of MCID Determinations

MCID studies aim to find a unique threshold value, but different methods produce a variety of MCID values. Anchor-based methods will produce different MCIDs depending on the criterion scale and the arbitrary selection or grouping of scale levels. Combining levels on a scale is a common but arbitrary procedure in MCID studies. Distribution-based methods also yield different values of MCID depending on the measure of statistical variability. Although methods relying on the SEM and MCD ensure the statistical soundness of an MCID value, other methods do not. More importantly, distribution-based approaches do not address the question of clinical importance and ignore the purpose of the MCID, which is to distinctly separate clinical importance from statistical significance. Another limitation of distribution-based approaches is that they are sample-specific, in the sense that the MCID value depends on the variability of the scores in the studied samples.

Several other factors can also influence the variability in reported MCID scores, including the characteristics of the study population. Patient-specific attributes such as age, sex, BMI, disease type and severity, treatment modality, and follow-up duration can notably impact the determined MCID score. Consequently, MCID scores should be viewed as context-specific rather than absolute values [18,32]. For example, in the case of a surgical procedure with high risk and prolonged recovery, patients would anticipate a more substantial improvement to consider it clinically relevant and justifiable compared to instances where minor lifestyle adjustments suffice.

In a recent study, Franceschini et al., 2023, assessed and compared the MCID values for a patient-reported outcome measure (PROM) in knee osteoarthritis patients treated with intra-articular platelet-rich plasma. Utilizing various calculation methods, the study found that the MCID values varied widely, ranging from 1.8 to 25.9 points. Anchor-based approaches produced values ranging from 6.3 to 25.9, while distribution-based methods were between 1.8 and 13.8 points. This study demonstrated how different MCID calculation methods result in markedly different values, substantially impacting the percentage of patients meeting the MCID in a specific population [33].

#### 4.6.2. Lack of Consideration for the Cost–Benefit Ratio

The cost of treatment is often neglected in determining the MCID. While the original definition of MCID acknowledged the need to consider costs (“in the absence of … excessive cost”), most studies rely on global assessment scales, which fail to account for the expenses associated with the change. Patients may perceive an improvement, but considering the costs, they might not find the benefit to be worth it [34].

#### 4.6.3. Challenges of Ordinal Scales in MCID Estimation

Ordinal scales are scales that rank items in order, but the distance between items on the scale is not necessarily equal. For example, a pain scale from 0 to 10 is an ordinal scale, but the difference between 2 and 3 on the scale may not be the same as the difference between 7 and 8 [35]. Using ordinal scales to estimate the MCID can be problematic. This is because the lack of a fixed unit in ordinal scales makes it difficult to compare changes in scores across different patients and different studies. Modern techniques like the Rasch model can help to transform ordinal scales into interval-based scales. Interval-based scales are scales where the distance between items on the scale is equal. This makes it easier to compare changes in scores on interval-based scales. However, the Rasch model is a complex statistical technique, and it is not always possible to use it to transform ordinal scales into interval-based scales. In addition, even if the Rasch model is used, it is important to interpret the results carefully, as the MCID is still a subjective measure.

#### 4.6.4. Changes in PRO Scores Are Linked to Baseline Scores

- Patients with higher baseline scores tend to show greater improvement [12,14].

Reasons for this issue:

a. Regression to the mean: extreme scores at baseline tend to move towards the average at follow-up.
b. Floor and ceiling effects: scores near the ends of the scale cannot show large changes;
c. Non-interval scales: the meaning of change is not the same across all points on the scale [15,36].

Addressing this issue:

a. Statistical control: this can eliminate the effect of baseline scores, but it may also mask true variation [37];
b. Percent change: this can account for differences in baseline scores, but it can be affected by floor and ceiling effects [38];
c. Range of MCID values: this can account for the fact that the meaning of change is not the same across all points on the scale.

Additional points:

d. Percent change scores can correct for high baseline scores when high scores indicate a worse health status;
e. Defining a range of MCID values instead of a single value can account for the fact that the meaning of change differs across the scale [12,14].

Determining the MCID in stroke patients can be challenging, as the impact of stroke can vary greatly depending on the severity, location, and type of stroke, as well as the patient’s age, medical history, and overall health. The MCID can also change over time as patients recover or experience further decline. The majority of studies in the literature reporting on MCID measures in stroke use various scales for assessing recovery after stroke. In addition, some studies have also described MCIDs related to revascularization after reperfusion therapy (Table 1 and Table 2).

## 5. Overview of MCIDs in Scales Reported in Stroke Research

In the following section, we discuss the commonly used scales in stroke for which an MCID has been estimated in the literature. Subsequently, we also examine the MCID of various other less commonly utilized scales in brief.

### 5.1. Modified Rankin Scale (mRS)

The Modified Rankin Scale (mRS), a seven-level, clinician-reported measure of global disability, is the most widely employed outcome scale in acute stroke trials [75]. It was first introduced in 1957 by John Rankin to measure the level of disability in patients with stroke. The mRS is a seven-point scale ranging from 0 to 6, with 0 representing no symptoms and 6 indicating death. The score reflects the level of disability, with higher scores indicating greater disability. The elements of the mRS are as follows: (1) no symptoms at all; (2) no significant disability, but there may be slight symptoms such as weakness or numbness; (3) slight disability, but able to carry out daily activities independently; (4) moderate disability, requiring some help with daily activities; (5) moderately severe disability, requiring assistance with most daily activities; (6) severe disability, bedridden and requiring constant nursing care; and (7) death [76,77]. One of the strengths of the mRS is its simplicity and ease of use. It can be completed quickly by clinicians or researchers and does not require specialized training or equipment. Additionally, the mRS has good inter-rater reliability and validity [78,79].

In their 2017 study, Cranston et al. estimated the MCID for mRS using expert opinions from 122 academic stroke neurologists. Based on a Delphi method, a change of one step anywhere along the seven-level Modified Rankin Scale (mRS) was considered to be clinically meaningful in stroke patients [54].

### 5.2. Barthel Index (BI)

The Barthel Index (BI) is a tool used to assess a person’s capability to manage activities of daily living (ADLs). It comprises 10 items that assess fundamental ADLs such as eating, grooming, bathing, dressing, bowel and bladder care, using the toilet, ambulating, carrying objects, and climbing stairs. The time taken and physical assistance required to perform each item are used in determining the assigned value of each item. Higher scores on the BI indicate better functional ability and independence in ADLs; each item is scored from 0 to 20, with maximum score of 100 [80].

Hsieh et al., 2008, evaluated the MCID of the Barthel Index (BI) in stroke patients. The study was conducted on a sample of stroke patients (mean duration since stroke onset: 70.4, 64.1 days (anchor method), and 1197.1, 1281.8 days (distribution method)). Three methods were used to estimate the MCID: anchor-based, distribution-based, and a combination of both methods. In the anchor-based method, patients’ global ratings of their activities of daily living function on a 15-point Likert-type scale were used as the anchor and in the distribution-based method, one SEM was used to estimate the MCID. The MCID for the BI was estimated to be five points (anchor-based), four points (distribution-based method), and four to five points (combination of both methods). The authors concluded that the MCID for the BI in stroke patients was estimated to be four to five points [73].

### 5.3. Reperfusion Therapy (Substantial Reperfusion (TICI 2b-3))

In the past decade, there have been significant advancements in the reperfusion therapies that are used to treat acute ischemic stroke during the first few hours of symptom onset [81]. Endovascular therapy (EVT) has become the standard of care for ischemic stroke caused by major artery obstruction up to 24 h after onset with the release of landmark trials in 2015 and 2018 [82]. Only one study till now has evaluated the MCID related to reperfusion therapy (substantial reperfusion (TICI 2b-3)) [46].

In their 2020 study, Lin et al. aimed to determine the MCID for substantial reperfusion, which was measured using the thrombolysis in cerebral infarction (TICI) score 2b-3 within three passes in non-inferiority clinical trials for acute ischemic stroke. A survey of 58 international neurointerventional and non-interventional vascular neurologist investigators was conducted. The survey involved assessing the MCID based on clinical scenario-based judgment. The results showed that the median MCID for substantial reperfusion was 3.1–5%, with an interquartile range of 1.1–3% to 5.1–10%. The distribution of the MCID was found to be similar between neurointerventionalists and non-interventional vascular neurologists [46] (Table 1 and Table 2).

### 5.4. Fugl-Meyer Assessment of the Upper Extremity (FMA-UE) and Fugl-Meyer Assessment of the Lower Extremity (FMA-LE)

The FMA-UE and FMA-LE are some of the most widely recognized measures of upper and lower extremity motor impairment in poststroke patients, respectively, with excellent inter-rater reliability [83]. The FMA-UE consists of five domains, including motor, sensory, balance, range of motion, and pain, each comprising multiple items scored on a three-point ordinal scale (0 = cannot perform, 1 = performs partially, 2 = performs fully). The motor section of FMA-UE assesses various aspects of movement, such as reflex, coordination, and speed. The FMA-UE is scored out of 66, with subscores of 36 for the upper arm and 30 for the wrist and hand. FMA-UE consists of 33 items in all, including 15 items related to aspects of movement for the upper arm (FMA-UA) and 12 items related to aspects of movement for the wrist and hand (FMA-W/H) [84,85,86]. The FMA-LE scale is commonly used to evaluate the recovery of lower limb motor function in stroke rehabilitation research. It comprises six items, each with multiple components, for a total of seventeen components. The six items of the FMA LE include reflex activity, flexor and extensor synergies, movement combinations, spasticity, coordination, and walking. The scale uses a three-point ordinal scoring system ranging from 0 (no performance) to 2 (faultless performance) for each component. The total score ranges from 0 (no motor function) to 34 (good motor recovery). They can be used to evaluate the effectiveness of rehabilitation interventions designed for improving upper and lower extremity function in stroke patients [87].

Four studies [48,56,63,64] evaluated the MCID in the FMA-UE, while one study [59] evaluated the MCID in the FMA-LE.

Hiragami et al., 2019, used an anchor-based method to determine the MCID in the FMA-UE at a mean of 49.4 ± 22.2 days post stroke (moderate to severe hemiparesis), with patient responses on a seven-point Likert scale as external criterion (anchor). Patients followed their usual rehabilitation regimen. The estimated MCIDs of the FMA-UE were 12.4 points for the upper extremity, 4.9 points for the wrist/hand, and 5.6 points for the upper arm [48].

Lundquist et al., 2017, estimated the MCID on 50 acute to subacute stroke patients (mean duration: 13.7, 9.0 days), using the patients’ global rating of change (GROC) on a seven-point Likert scale at re-test as an anchor, at a mean of 22.0 ± 4.1 days from inclusion. Patients followed their usual rehabilitation regimen. The MCID of the FMA-UE was derived to be a four-point change [56].

Arya et al., 2011, estimated the MCID of the FMA-UE on 71 poststroke (mean duration since stroke onset: 8.42 weeks) patients using anchor-based methods with the mRS and global rating of patient-perceived changes (GRPPC) as anchors. The MCID was assessed at 4 weeks post intervention (Meaningful Task Specific Training (MTST) or physical and neurodevelopmental therapy proposed by Brunnstorm and Bobath). The MCID for the FMA-UE were found to be 9 points and 10 points anchored to the mRS and GRPPC, respectively [64].

Page et al., 2012, estimated the MCID of the FMA-UE in 160 individuals with stable, mild-to-moderate upper extremity hemiparesis (mean duration since stroke onset: 59.37, 63.22 months). An anchor-based method was used with each patient’s self-reported degree of upper extremity motor improvement on a global rating of change (GROC) scale as the anchor. Participants received 26 days of intervention (repetitive task-specific training (RTP) regimen with electrical cortical stimulation using the Northstar Stroke Recovery System (Northstar Neuroscience Inc, Seattle, Washington)). Depending on the various aspects of limb movement, the MCID of the FMA-UE ranged from 4.25 to 7.25 points [63].

Pandian et al., 2016, estimated the MCID of the FMA-LE on 65 poststroke patients with hemiparesis (mean poststroke duration = 16.42 months) who underwent conventional motor therapy to the lower limb based on neurophysiological approaches for 10 weeks. The MCID for the FMA-UE was found at six points using an anchor-based method with the GRPPC as anchor [59].

Thus, as per the current literature, the overall MCID for the FMA-UE and LE has been estimated to range from 4 to 12.4 points, meaning that an increase in FMA-UE and LE score by 4 to 12.4 points is considered to indicate a meaningful improvement in upper/lower extremity function (Table 1 and Table 2).

### 5.5. Gait Speed

Gait speed is used as a measure to evaluate mobility and assess recovery from stroke. The MCID for gait speed in stroke patients can vary depending on the study and population being evaluated [88,89].

Martín-San Agustín et al., 2021, assessed the MCID of gait speed for community ambulation in 111 poststroke patients at various stages of rehabilitation and severity levels (mean duration since stroke onset: 51.8 ± 31.5 days). Gait speed was measured using the 4MGS (4 m gait speed) test, which involved marking out a 4 m course in a clinical assessment room using tape. Patients were asked to complete the walk at their most comfortable speed, and the time was recorded using a stopwatch. Timing began after the patient had the opportunity to accelerate and stopped when the patient’s first foot completely crossed the 4 m line. Subjects performed two trials, and the faster time was recorded. Participants who were unable to complete the test were given a gait speed score of 0 m/second (m/s). The MCID for gait speed was determined as 0.21 m/s and 0.11 m/s at 4 weeks and 0.19 m/s and 0.04 m/s at 8 weeks for community ambulators and household ambulators, respectively [41].

Bohannon et al., 2016, estimated the MCID of comfortable gait speed in 35 poststroke patients (mean duration from stroke onset: 27.2 days) using an anchor-based method. The lowering of two or more levels of gait assistance necessary over the course of rehabilitation served as the anchor. Patients’ gait speed was assessed by measuring the time it took for them to walk a 20-foot distance at a comfortable pace using a digital stopwatch. Timing was initiated after patients had the chance to accelerate. An improvement in gait speed of 0.13 m/s or more was found to be clinically important among the participants [62].

Fulk et al., 2011, estimated the MCID of gait speed in 44 stroke survivors (mean duration from stroke onset: 138.6 (74.5) days) using two different anchors: stroke survivors’ and physical therapists’ perceptions of change in walking ability (15-point ordinal global rating of change (GROC) scale). Participants’ self-selected gait speed was measured at the beginning and end of outpatient physical therapy using a GAITRite walkway (CIR Systems, Inc., Havertown, PA, USA). GAITRite is an electronic walkway equipped with pressure sensors that records footfall pattern data and calculates various aspects of gait, including speed, step length, and percentage of gait cycle in single limb stance. Participants were asked to walk across the 17-inch × 3-inch electronic walkway at their own pace with any assistive devices or orthotics they were prescribed and with any necessary assistance or supervision to ensure safety. The use of assistive devices and orthotics was not controlled during the study, and only one walking trial was recorded. GAITRite is considered a valid and reliable method of assessing gait [90]. Depending on the anchor, the estimated significant change in gait speed varied from 0.190 m/s (physical therapists’ perception) to 0.175 m/s (participants’ perception) [67].

Tilson et al., 2010, estimated the MCID of comfortable gait speed (CGS), with an improvement in the Modified Rankin Scale (mRS) score as the anchor, in 283 stroke patients between 20 and 60 days of stroke onset. The study used a standardized procedure to measure gait speed, specifically the 10-meter walk test (10mWT). The walking course for the test was 14 m long, consisting of a 2 m warm-up, 10 m used for speed measurement, and 2 m for slowing down to a stop. Participants were instructed to walk at a comfortable pace and were allowed maximum assistance by one person for balance and stability but not for paretic limb advancement. Participants used their most commonly used assistive device or orthotic device at each time point, such as a cane or walker or ankle–foot orthosis. Two trials were conducted in succession, and participants were allowed to rest between trials as needed while seated or standing. The MCID was calculated as a 0.16 m/s increase in CGS that was anchored to the mRS [70].

Details about the MCIDs of various other scales, like the Mini-Balance Evaluation Systems Test (Mini-BESTest), Five-Repetition Sit-to-Stand Test (5STS), Knee Range of Motion (ROM), Wisconsin Gait Scale (WGS), Arm Motor Ability Test (AMAT), Stroke Rehabilitation Assessment of Movement (STREAM), Stroke Impact Scale-16 (SIS-16), Nottingham Extended Activities of Daily Living (NEADL) scale, and a few others, are provided in the Supplementary Material.

## 6. Applying the MCIDs Reported in this Review to Completed Clinical Trials

A search was conducted to identify trials that used the same scales to evaluate the effectiveness of stroke prevention, treatment, and rehabilitation therapy as those reported in this review. The difference in scale score between the intervention and control groups which was deemed significant in these trials was then compared with the MCID thresholds reported in the literature and presented in this review to provide a possible impact on trial interpretation using the MCID (Table 3).

## 7. Discussion

This review article provides an overview of the MCID for various scales that have been used in stroke research to measure the smallest clinically meaningful changes in outcome measures. This review is significant in that it combines all MCID scales currently reported in stroke patients, providing a comprehensive resource for future trials. By examining the different MCID scales, researchers can better understand which scales are most appropriate for their particular study and outcome measures. Future researchers should emphasize the importance of considering clinical relevance when interpreting study results. Statistical significance alone does not necessarily indicate clinical relevance and MCID scales can help bridge this gap [54].

When conducting clinical research trials, it is important to ensure that the method used to determine the outcome should also be appropriate and reliable in order to ensure that the primary outcome is meaningful for patients. Also, the nature of the scale used is an important consideration when determining the MCID. If the scale is ordinal, it may not be appropriate to use the raw score difference as the MCID because the distance between each category may not be equal. In such cases, the Rasch model can be used to transform the ordinal scale into an interval-based scale, allowing for a more accurate determination of the MCID [7].

Like multiple other disorders, determining the MCID in stroke research is not a straight-forward process, as it can vary depending on the outcome measure, population, and clinical context [119]. Recently, Goyal et al. reviewed the difficulties with the MCID, sample size, and practical issues that researchers confront while designing acute stroke trials, and they provided a paradigm for developing meaningful stroke trials that have the potential to alter clinical practice. One of the primary challenges in applying the MCID to clinical trials is that the concept of the MCID is still vague and lacks clear guidelines for definition. Additionally, practical and financial constraints, rather than the MCID, often dictate the sample size of a trial. In certain instances, after determining the largest practicable sample size, researchers specify the anticipated treatment effect and MCID, which could result in biased assumptions. Hence, Goyal et al. suggested that an external multidisciplinary committee should be set up to decide upon the MCIDs for key outcome measures, and the stroke research community needs to work towards establishing a clear pathway for conducting trials with a sample size that is large enough to detect the MCID with appropriate power [8].

While the Modified Rankin Scale (mRS) is a commonly used outcome measure in stroke research, our literature review has revealed that no studies have evaluated the MCID of the mRS in stroke per se. Studies carried statements like “a single-point change on the mRS is clinically relevant” [120], “a single-point change on the mRS will always be clinically relevant” [121], “we arbitrarily defined ‘important change’ as the change of one grade on mRS” [61], “participants who demonstrated a change on the MRS of 1 or greater were considered to have an important amount of change” [91], or “sensitivity to clinically meaningful change has been established for shifts of mRS scores of ≥1” [92], which were not backed by calculations or scientific evidence. In a study by Cranston et al., 2017, a one-level change was considered as clinically relevant based on the clinical judgement of neurologists obtained by survey [54]. The lack of robust studies evaluating the MCID of the mRS in stroke research reveals a significant gap in the literature. Further studies are needed to establish a clear understanding of what constitutes a “minimal” clinically meaningful change in mRS score in stroke research.

Incorporating patient perspectives is crucial when estimating the MCID, as it helps to determine what constitutes a meaningful difference in patient-reported outcomes [8,30]. However, in the present review, none of the included studies explicitly reported on whether they included patient perspectives in estimating the MCID (Table 1). Especially when using anchor-based methods, none of the studies included in this review mentioned how many of the participants providing perspectives on Likert-type scales were patients (Table 1). Additionally, in the three studies that used the Delphi method (survey), all the participants who provided perspectives on the MCID were experts (physicians or therapists) and patients were not involved (Table 1).

Moreover, in the context of stroke trials, the issue of proxy responses in patient-reported outcome measures (PROMs) is an important consideration that has implications for the usefulness of the MCID. In a previous systematic review of caregiver responses on cancer-related PROMs, group-level patient–proxy concordance was found to be generally good for multidimensional HRQoL (Health-Related Quality of Life) tools, but proxies consistently underestimated patient QoL and physical and emotional function compared to patients’ estimation of these outcomes. Although good concordance was promising, there was still substantial residual variability that needed to be minimized through appropriate adjustment factors [122]. None of the studies incorporated in the present review provided explicit details on whether proxy respondents were allowed to provide their perspectives on PROMs or Likert-type scales that captured the patients’ perception of change, revealing a noteworthy gap in the literature (Table 1). This raises concerns about the under-recognition of this critical aspect among researchers involved in estimating the MCIDs of stroke-related scales. In stroke trials, the use of proxies to respond on behalf of patients in PROMs may lead to an underestimation of the true effect of an intervention on patient outcomes, potentially leading to an over-reliance on MCID thresholds that are not truly representative of patient perspectives. Therefore, while the use of proxy responses in PROMs may be necessary in some cases, additional work is needed to identify adjustment factors that can help minimize the residual variability caused by proxy responses and to determine which tools have the strongest evidence base for concordance and adjustment factors [122,123].

These are significant gaps in the literature that raise questions about the relevance of the reported MCID values and we will not truly understand the degree of clinical improvement that may be impactful for stroke survivors if their perspective is never incorporated. Future research should aim to incorporate patient perspectives when estimating MCID, and if not, then whether the “patient-proxy” or “proxy-proxy” perspective was used should be clearly documented by the investigators. By doing so, stroke trials can better capture patient perspectives and improve the usefulness of MCID thresholds in evaluating the effectiveness of interventions.

The application of MCIDs to completed clinical trials revealed that the majority of studies demonstrated consistency between statistical significance and clinical relevance, while a few exhibited discrepancies in this regard. It is crucial to interpret these findings with caution, as the determination of the MCID is context-specific and varies depending on several factors, including poststroke duration, type of therapy, measurement tools, clinical setting, and side of paresis. Nonetheless, future researchers may consider integrating the MCID into sample size estimation for clinical trials, thereby ensuring trials are not only statistically significant but also clinically relevant.

The Stroke Therapy Academic Industry Roundtable (STAIR) consortium has put forth several suggestions for methodological enhancements in future research. These include the use of multimechanism drugs, the integration of new interventions with thrombolytics, and the application of advanced imaging techniques for patient selection. However, trials that have followed these methodologies have not yielded beneficial results, indicating the need for more stringent preclinical designs before progressing to phase 2 or 3 randomized controlled trials (RCTs) [124,125].

The Stroke Preclinical Assessment Network (SPAN) has proposed measures to improve the quality of experimental studies. New compounds should be evaluated in rigorous multicenter preclinical randomized blinded studies, similar to clinical trials. These studies should involve multiple species and use clinically relevant outcomes within appropriate time windows. This approach could potentially enhance the translational value of preclinical stroke research [126].

In addition to these, the concept of the MCID could be integrated into the experimental design of animal studies. For instance, determining the MCID for different outcome measures in animal models of stroke could help in quantifying the effectiveness of various interventions. The significant individual differences among animals pose a unique challenge, and the following suggestions can be used to address this concern:

▪ Defining the MCID in animal experiments: identify animal outcome measures closely paralleling clinical MCIDs, focusing on functional outcomes relevant to human stroke, such as motor skills, cognition, and behavior;
▪ Considering statistical approaches: employ statistical methods like mixed-effects models and within-subject designs to account for individual variability;
▪ Increasing sample sizes: recruit larger animal cohorts to enhance statistical power and better represent population variability;
▪ Utilizing homogeneous animal models: select animal models with reduced genetic and phenotypic diversity to minimize variability;
▪ Prioritizing biological relevance: choose animal models that closely mimic human stroke pathophysiology and recovery processes;
▪ Incorporating longitudinal assessments: track animal outcomes over time to capture meaningful changes and assess the durability of effects;
▪ Collaborating with clinicians: engage with clinical experts to ensure animal research aligns with clinically relevant MCIDs and patient-centered outcomes [127,128,129,130,131].

By integrating these strategies, the translation of MCIDs into basic stroke research can be strengthened, fostering more clinically meaningful and impactful preclinical studies. Furthermore, the application of MCIDs in basic research could also involve a bilateral flow from bench to bedside and back to the bench [132]. This would mean not only applying the concept of MCID to animal studies but also using the findings from these studies to refine the MCID in clinical practice.

Overall, this review highlights the need for standardized and validated MCID scales in stroke research to help clinicians and researchers interpret study results and to make informed decisions about patient care. The use of MCID scales can also help to ensure that future stroke trials are designed and conducted in a way that is most meaningful and relevant to patients.

The present review has some limitations. This review may have missed unpublished studies or those published in languages other than English, which could lead to publication bias and affect the validity of the conclusions. This review included studies with different study designs, sample sizes, outcome measures, and follow-up durations, which could result in a heterogeneity of the results and limit the generalizability of the findings.

## 8. Conclusions

In conclusion, the MCID is an important concept in stroke research that helps to evaluate the clinical significance of treatment effects. Determining the MCID for different outcome measures requires a careful consideration of various factors, and different methods can be used to estimate the MCID. Ultimately, a clear comprehension of the MCID can potentially enhance the management of stroke patients.

## Figures and Tables

**Table 1:** Demographic characteristics, methods, and MCID thresholds in the various scales reported by studies in the literature.

Serial no.	Author, Year	Design	Patients by Anchor/ Distribution/ Delphi (Total)	Mean Age	Disease	Specific Treatment	Scale	Subscale/ Dimension	Type of Scale (Clinician- or Patient-Reported). Were Proxies Permitted to Respond on Behalf of Patients?	Anchor Based	In the Anchor Method, What No. of Participants Who Provided Perspectives on Likert-Type Scales Were Patients?	Distribution-Based	Delphi Method	Delphi Method: How Many in the “Expert” Groups Were Patients with Illness?	MCID Thresholds
1	Tamura et al., 2021 [39]	Multi-centric, retrospective study	80/-/- (80)	-	Subacute stroke	-	Berg Balance Scale (BBS)	x	BBS: clinician-reported scale. Consent not obtained from the patients (citing it as retrospective study). Likely all patients reported on the anchors using GROC on a 15-point Likert scale. No information (NI) on proxies.	Yes	Likely that all 80 participants who provided perspectives on the Likert-type scale (GROC) were patients.	x	-	N/A	Assisted walking group: 5 points; unassisted walking group: 4 points
2	Beauchamp et al., 2021 [40]	Prospective cohort	50/50/- (50)	60.8 (9.4)	Stroke	Rehabilitation	Mini-Balance Evaluation Systems Test (Mini-BESTest)	x	MiniBESTest: clinician-reported scale. Consent was obtained from all the patients. Likely that all patients reported on the anchors using GROC on a Likert scale. NI on proxies.	Yes	Likely that all 50 participants who provided perspectives on the Likert-type scale (GROC) were patients.	Yes	-	N/A	4 points
3	Agustin et al., 2021 [41]	Prospective cohort	111/-/- (111)	68.3 (12.1)	Stroke	Rehabilitation	Five-Repetition Sit-to-Stand test (5STS) in seconds, Gait speed in m/s	x	Gait speed and 5STS: both clinician-reported scales. Consent was obtained from all the patients. Likely that all patients reported on the anchors using GROC on a Likert scale. NI on proxies.	Yes	Likely that all 50 participants who provided perspectives on the Likert-type scale (GROC) were patients.	x	-	N/A	5STS at 8 weeks: all patients: 0.76, household limited: 0.72, limited community: 3.09; at 4 weeks: all patients: 1.18, household limited: 1.9, limited community: 2.92. Gait speed at 8 weeks: all patients: 0.09, household limited: 0.04, limited community: 0.11; at 4 weeks: all patients: 0.19, household limited: 0.19, limited community: 0.21
4	Fu et al. [42]	Secondary analysis of RCT	400/400/- (400)	72.0 (12.5)	Stroke	-	Physical Component Summary (PCS) score of the Short Form 36 (SF-36)	x	SF-36: patient-reported outcome measure (PROM). Consent was obtained from all the patients. Likely that all patients reported on the anchors using the PCS SF-36 scale. NI on proxies.	Yes	Likely that all 400 participants who provided perspectives on the PCS SF scale (Likert-type scale) were patients.	Yes	-	N/A	1.8 to 3.0 units
5	Guzik et al., 2020 [43]	Prospective cohort	50/50/- (50)	60.9 (11.2)	Stroke	Rehabilitation	Knee range of motion (ROM)	x	MiniBESTest: clinician-reported scale. Consent was obtained from all the patients. Likely that all patients reported on the anchors using GROC on a Likert scale. NI on proxies.	Yes	Likely that all 50 participants who provided perspectives on the Likert-type scale (patient perception of improvement) were patients.	Yes	-	N/A	Affected side: 8.48 degrees, unaffected side: 6.81 degrees
6	Alzyoud et al., 2020 [44]	Prospective cohort	43/43/- (43)	71.6 (11.4)	Stroke	Rehabilitation	Sitting Balance Scale (SBS), Function in Sitting Test (FIST)	x	SBS and FIST: both clinician-reported scales. Consent was obtained from all the patients. Likely that all patients reported on the BI as anchor. NI on proxies.	Yes	Likely that all 50 participants who provided perspectives on the Barthel Index were patients.	Yes	-	N/A	SBS: 5, FIST: 4
7	Everton et al., 2020 [45]	Post hoc analysis of previous RCT (STEPS); Survey	154/154/84 (238)	-	Stroke	Rehabilitation	Dysphagia Severity Rating Scale (DSRS)	x	DSRS: clinician-reported scale. Consent was obtained from all the patients (or surrogates). Likely that all patients reported on the anchors (anchor/Likert scale used is not mentioned). NI on proxies.	Yes	No information on the anchor used.	Yes	Yes	No explicit information on how many were patients but all were probably SLTs.	Anchors: aspiration at week 2: 2.5 points; oral vs. non-oral feeding at week 2: 1.0; 0.5 SD: 1.9, SEM 0.3. Survey: 1 point
8	Lin et al., 2020 [46]	Survey	-/-/58 (58)	-	Stroke	Endovascular thrombectomy	Substantial reperfusion (TICI 2b-3)	x	Substantial reperfusion (TICI 2b-3): clinician-reported outcome. No patients involved in the survey.	x	Not applicable.	x	Yes	None were patients.	Median: 3.1–5%
9	Chen et al., 2019 [47]	Prospective observational study	-/115/- (115)	54.2 (11.1)	Stroke	Rehabilitation	Modified Ashworth Scale (MAS)	x	MAS: clinician-reported scale. Consent was obtained from all the patients. Only distribution-based method used. Likely that all patients reported on a Likert scale. NI on proxies.	x	Not applicable.	Yes	-	N/A	0.5 SD: upper extremity: 0.48, lower extremity: 0.45. 0.8 SD: upper extremity: 0.45, lower extremity: 0.73
10	Hiragami et al., 2019 [48]	Post hoc analysis of a previous RCT	12/-/- (12)	67.8 (10.5)	Stroke (moderate to severe hemiparesis)	Rehabilitation	Fugl-Meyer assessment of the upper extremity (FMA-UE)	Motor	FMA UE: clinician-reported scale. Consent was obtained from all the patients. Likely that all patients reported on the anchors using GROC on a Likert scale. NI on proxies.	Yes	Likely that all 12 participants who provided perspectives on the Likert-type scale. (GROC) were patients.	x	-	N/A	Upper extremity: 12.4; upper arm: 5.6; wrist/hand: 4.9
11	Guzik et al., 2019 [49]	Prospective observational study	50/50/- (50)	60.9 (11.2)	Stroke	Rehabilitation	Wisconsin Gait Scale (WGS)	x	WGS: clinician-reported scale. Consent was obtained from all the patients. Likely that all patients reported on the GROC using Likert scale. NI on proxies.	Yes	Likely that all 12 participants who provided perspectives on the Likert-type scale (GROC) were patients.	Yes	-	N/A	2.25
12	Wu et al., 2019 [50]	Prospective observational study	65/65/- (65)	53.5 (11.7)	Stroke	Rehabilitation	Montreal Cognitive Assessment (MoCA)	x	MoCA: clinician-reported scale. Consent was obtained from all the patients. Likely that all patients reported on the anchors using Stroke Impact Scale 3.0 (Likert-type scale). NI on proxies.	Yes	Likely that all 65 participants who provided perspectives on the Stroke Impact Scale 3.0 (Likert-type scale) were patients.	Yes	-	N/A	Anchor-based: 1.22 points; distribution-based: 2.15 points
13	Fulk et al., 2018 [51]	RCT	265/-/- (265)	61.3 (12.8)	Stroke	Rehabilitation	6-min walk test (6MWT)	x	6 MWT: clinician-reported scale. Consent was obtained from all the patients. Likely that all patients reported on the anchors using Stroke Impact Scale 3.0 (Likert-type scale.) NI on proxies.	Yes	Likely that all 265 participants who provided perspectives on the Stroke Impact Scale 3.0 (Likert-type scale) were patients.	x	-	N/A	71 m
14	Chen et al., 2018 [52]	Pooled data from three clinical trials	82/82/- (82)	55.3 (10.7)	Stroke	Rehabilitation	Arm accelerometer, tools used: clinical measurement tools: Motor Activity Log (MAL), Stroke Impact Scale (SIS), and Nottingham Extended Activities of Daily Living (NEADL).	x	SIS and MAL: patient-reported outcome measures (PROMs); NEADL: clinician-reported scale. Consent was obtained from all the patients. Likely that all patients reported on the anchors using Stroke Impact Scale 3.0 (Likert-type scale). NI on proxies.	Yes	Likely that all 82 participants who provided perspectives on Likert-type scale (SIS) were patients.	Yes	-	N/A	547–751 mean counts
15	Song et al.,2018 [53]	Prospective cohort	73/-/- (73)	63.94 (12.78)	Stroke	-	Berg Balance Scale (BBS)	x	BBS: clinician-reported outcome. Informed consent was obtained from all participants. Likely that all patients reported on the anchors using GROC on a 15-point Likert scale. NI on proxies.	Yes	Likely that all 73 participants who provided perspectives on the 15 GROC (15-point Likert scale) were patients.	x	-	N/A	12.5 points
16	Cranston et al., 2017 [54]	Survey	-/-/122 (122)		Stroke	Novel safe neuroprotective agent	Modified Rankin Scale (mRS), safe acute ischemic stroke treatment	x	mRS and percent change needed for safe acute ischemic stroke treatment: expert-reported change. Patients not involved.	x	Not applicable.	x	Yes	None were patients.	Modified Rankin Scale (mRS): 1 point; safe acute ischemic stroke treatment: 1.1–1.5%
17	New et al., 2016 [55]	Prospective cohort	366/366/- (366)	-	Stroke	Rehabilitation	de Morton Mobility Index (DEMMI)	x	DEMIM: clinician-reported scale. Consent was obtained from all the patients. Likely that all patients reported on the anchors using GROC on a Likert scale. NI on proxies.	Yes	Likely that all 366 participants who provided perspectives on the Likert-type scale (GROC) were patients	Yes	-	N/A	Anchor-based: 8.0; distribution-based: 2.9
18	Lundquist et al., 2017 [56]	Prospective observational study	50/-/- (50)	70.2 (10.1)	Stroke	-	Fugl-Meyer assessment of the upper extremity (FMA-UE)	Danish version	FMA UE: clinician-reported scale. Consent was obtained from all the patients. Likely that all patients reported on the anchors using GROC on a Likert scale. NI on proxies.	Yes	Likely that all 366 participants who provided perspectives on the Likert-type scale (GROC) were patients.	x	-	N/A	≥4
19	Fulk et al., 2017 [57]	Secondary analysis of data from the Everest RCT	146/146/- (146)	57.1 (11)	Stroke	Rehabilitation	Arm Motor Ability Test (AMAT)	x	AMAT: clinician-reported scale. No information provided regarding consent. Likely that all patients reported on the anchors using GROC on a Likert scale. NI on proxies.	Yes	Likely that all 146 participants who provided perspectives on the Likert-type scale (GROC) were patients.	Yes	-	N/A	≥0.44 points
20	Correa et al., 2017 [58]	Prospective cohort	-/20/- (20)	55.2 (9.9)	Stroke	Rehabilitation	Gait Deviation Index (GDI)	x	GDI: clinician-reported scale. Not applicable, only distribution-based method used.	x	Not applicable; only distribution-based method used.	Yes	-	N/A	Non-paretic limb: 9.4; paretic limb: 7.5
21	Pandian et al., 2016 [59]	Prospective observational study	65/-/-	44.2 (12.8)	Stroke	Rehabilitation	Fugl-Meyer assessment: Lower extremity (FMA-LE)	x	FMA LE: clinician-reported scale. Consent was obtained from all the patients. Likely that all patients reported on the anchors using GRPPC on a Likert scale. NI on proxies.	Yes	Likely that all 65 participants who provided perspectives on the Likert-type scale (GRPPC) were patients.	x	-	N/A	6
22	Chen et al., 2016 [60]	Prospective cohort	65/65/-	52.8 (11.6)	Stroke	Rehabilitation	EuroQoL 5-Dimensions Questionnaire (EQ-5D-5L); Visual analog scale (EQ-VAS)	x	EQ-5DQ, EQ-VAS: all are patient-reported outcomes (PROMs). Consent was obtained from all patients. Likely that all patients reported on the anchors using FAC and GRPPC on a Likert scale. NI on proxies.	Yes	Likely that all 65 participants who provided perspectives on the Likert-type scale (FAC and GRPPC) were patients.	Yes	-	N/A	EQ-Index: 0.1; EQ-VAS: 8.61–10.82
23	Kim et al., 2015 [61]	Prospective observational study	487/-/-	68.3 (8.1)	Stroke	-	EQ-5D, SF-36 v2	x	EQ-5DQ, SF 36 v2: all are patient-reported outcomes (PROMs). Consent was obtained from all the patients. Likely that all patients reported on the anchors using a 5-point Likert-type scale. NI on proxies.	Yes	Likely that all 487 participants who provided perspectives on the 5-point Likert-type scale were patients.	x	-	N/A	EQ-5D: 0.08–0.12; SF-6D 0.04–0.14
24	Bohannon et al., 2013 [62]	Retrospective cohort	35/-/-	-	Stroke	Rehabilitation	Comfortable gait speed	x	Gait speed: clinician-reported scale. Informed consent was waived as the study involved the secondary analysis of archived records. Likely that all patients reported on the anchors 5-point Likert-type scale. NI on proxies.	Yes	Likely that all 35 participants who provided perspectives on the 5-point Likert-type scale were patients.	x	-	N/A	Change in walking speed of 0.13 m/s
25	Page et al., 2012 [63]	RCT	146/-/- (146)	57.1 (11)	Stroke	Rehabilitation	Fugl-Meyer assessment of the upper extremity (UE-FM)	x	FMA-UE: clinician-reported scale. Consent was obtained from all the patients. Likely that all patients reported on the anchors using GROC on a 5-point Likert-type scale. NI on proxies.	Yes	Likely that all 146 participants on whom clinician rated 5-point Likert- scale (GROC) were patients.	x	-	N/A	4.25–7.25
26	Arya et al., 2011 [64]	RCT	71/-/- (71)	52.4 (9.5)	Stroke	Rehabilitation	Fugl-Meyer assessment of the upper extremity (UE-FM)	x	FMA UE: clinician-reported scale. Consent was obtained from all the patients. Likely that all patients reported on the anchors using GRPPC on a Likert-type scale. NI on proxies.	Yes	Likely that all 71 participants who provided perspectives on the Likert-type scale (GRPPC) were patients.	x	-	N/A	9 to 10
27	Wu et al., 2011 [65]	RCT	78/78/- (78)	54.3 (11.9)	Stroke	Rehabilitation	Nottingham Extended Activities of Daily Living (NEADL) scale	x	NEADL: patient-reported outcome measure (PROM). Consent was obtained from all the patients. Likely that all patients reported on the anchors using SIS 3.0 (a Likert-type scale.) NI on proxies.	Yes	Likely that all 78 participants who provided perspectives on the Likert-type scale (SIS 3.0) were patients.	Yes	-	N/A	6.1 points
28	Wang et al., 2011 [66]	Pooled data from three clinical studies	51/51/- (51)	55.3 (10.3)	Stroke	Robot-assisted training	ABILHAND questionnaire	x	ABILHAND: patient-reported outcome measure (PROM). Consent was obtained from all the patients. Likely that all patients reported on the anchors using SIS 3.0 (a Likert-type scale.) NI on proxies.	Yes	Likely that all 51 participants who provided perspectives on the Likert-type scale (SIS 3.0) were patients.	Yes	-	N/A	0.26 to 0.35
29	Fulk et al., 2011 [67]	Prospective cohort	44/-/- (44)	61.8 (14.7)	Stroke	Rehabilitation	Change in gait speed	x	Gait speed: clinician-reported outcome. Consent was obtained from all the patients. Likely that all patients reported on the anchors using GROC on a 7-point Likert-type scale. NI on proxies.	Yes	Likely that all 44 participants who provided perspectives on the 7-point Likert-type scale (GROC) were patients.	x	-	N/A	0.175 to 0.19 m/s
30	Lin et al., 2011 [68]	RCT	74/74/- (74)	57.1 (11.7)	Stroke	Rehabilitation	Stroke-Specific Quality of Life Scale (SS-QOL)	Mobility, self-care, UE function	SS-QOL: patient-reported outcome measure (PROM). Consent was obtained from all the patients. Likely that all patients reported on the anchors using a 5-point Likert-type scale. NI on proxies.	Yes	Likely that all 74 participants who provided perspectives on the 5-point Likert-scale were patients.	Yes	-	N/A	Mobility: 1.5–2.4; self-care: 1.2–1.9; UE function: 1.2–1.8
31	Fulk et al., 2010 [69]	Prospective cohort	36/36/- (36)	60.9 (15.6)	Stroke	Rehabilitation	Stroke Impact Scale-16 (SIS-16)	x	SIS-16: patient-reported outcome measure (PROM). Consent was obtained from all the patients. Likely that all patients reported on the anchors using a 5-point Likert-type scale. NI on proxies.	Yes	Likely that all 36 participants who provided perspectives on GROC scale were patients.	x	-	N/A	9.4–14.1
32	Tilson et al., 2010 [70]	Secondary analysis of the LEAPS RCT	283/-/- (283)	63.5 (12.5)	Stroke	Rehabilitation	Comfortable gait speed	x	Comfortable gait speed: clinician-reported outcome measure. Consent was obtained from all the patients. Clinicians reported on all participants using mRS change. NI on proxies.	Yes	Likely that all 283 participants who were assessed on mRS were patients.	x	-	N/A	0.16 m/s
33	Hsieh et al., 2008 [71]	Prospective cohort	81/81/- (81)	55.9 (13.3)	Stroke	Rehabilitation	Stroke Rehabilitation Assessment of Movement (STREAM) measure	Lower extremity, upper extremity, mobility	Barthel Index: clinician-reported outcome measure. Only patients or their proxies who gave informed consent participated in the study. But whether any proxies provided the rating on GROC scale is not provided. Likely that all patients reported on the anchors using a 5-point Likert-type scale (GROC).	Yes	Likely all 81 participants who provided perspectives on GROC (Likert-type scale) scale were patients.	x	-	N/A	UE: 2.2, LE: 1.9, mobility: 4.8
34	Lang et al., 2008 [72]	RCT	52/52/- (52)	64 (14)	Stroke	Constraint induced movement therapy	Grip strength, composite upper extremity strength, Action Research Arm Test (ARAT), Wolf Motor Function Test (WMFT), Motor Activity Log (MAL), duration of upper extremity use	Dominant and non-dominant hand	Grip strength, composite upper extremity strength, Action Research Arm Test (ARAT), Wolf Motor Function Test (WMFT), Motor Activity Log (MAL), duration of upper extremity use: Clinician-reported outcome measure. Consent was obtained from all the patients. Likely that all patients reported on the anchors using GRPPC scale. NI on proxies.	Yes	Likely that all 81 participants who provided perspectives on GRPPC (5-point Likert scale) were patients.	x	-	N/A	Dominant hand: grip strength: 5.0 Kg, ARAT: 12 points, WMFT: 1.0 points, MAL score: 1.0. Non-dominant hand: grip strength: 6.2 Kg, ARAT: 17 points, WMFT: 1.2 points, MAL: 1.1 points
35	Hsieh et al., 2007 [73]	Prospective cohort	81/-/- (81)	55.9 (13.3)	Stroke	-	Barthel Index (BI)	x	STREAM: clinician-reported outcome measure. Only patients or their proxies who gave informed consent participated in the study. But whether any proxies provided the rating on GROC scale is not provided. Likely that all patients reported on the anchors using a 15-point Likert-type scale.	Yes	Likely that all 81 participants who provided perspectives on 15-point Likert scale were patients.	-	-	N/A	1.85
36	Beninato et al., 2006 [74]	Case series	113/-/- (113)	-	Stroke	-	FIM instrument	Total, motor, cognitive	FIM: clinician-reported outcome. No information regarding consent provided in manuscript. Likely that all patients reported on the anchors using GROC on a 15-point Likert scale. NI on proxies.	Yes	Likely that all 113 participants who provided perspectives on GROC on a 15-point Likert scale were patients.	x	-	N/A	Total: 22, motor: 17, cognitive: 3

**Table 2:** Anchors, viewpoints, statistical methods, and the type of method used to arrive at the respective MCID thresholds.

Serial No.	Author	Year	Anchor-Based Methods					Distribution-Based Methods		Delphi Method/From Surveys
			Number of Anchors	Anchor(s)	Viewpoint	Cutoffs Used	Statistical Methods	Number of Distribution Criteria Used	Distribution Criteria	
1	Tamura et al., [39]	2021	1	Functional Ambulation Categories (FACs)	Clinician	FAS change ≥ 1 point	ROC	x	x	x
2	Beauchamp et al., [40]	2021	1	Global rating of change	Clinician	Response on a scale	Mean change approach, ROC	1	SEM	x
3	Agustin et al., [41]	2021	1	Global rating of change	Patient	Response on a scale (15-point Likert scale)	Median change (within patient change), ROC	x	x	x
4	Fu et al. [42]	2021	2	Perceived Health Change	Patient	Response on a scale (Physical Component Summary (PCS) score of the Short Form 36 (SF-36), range 0 to 100)	Linear regression analysis, root mean square error (RMSE) ANOVA	1	0.2 SD	x
5	Guzik et al., [43]	2020	1	Patients’ perception of improvement	Patient	No change vs. improvement vs. worsening	Mean change, regression analysis, ROC	1	SEM	x
6	Alzyoud et al., [44]	2020	1	Barthel Index (BI)	Clinician	BI ≥ 2 vs. <2	ROC	1	Effect size, SEM	x
7	Everton et al., [45]	2020	x	Aspiration at week 2 and oral vs. non-oral feeding at week 2	Clinician	Penetration Aspiration Score (PAS) ≥ 3	Mean change	2	0.5 SD, SEM	1
8	Lin et al., [46]	2020	x	x	x	x	x	x	x	Median: 3.1–5%
9	Chen et al., [47]	2019	x	x	x	x	x	2	0.5 SD, 0.8 SD	x
10	Hiragami et al., [48]	2019	1	Global rating of change	Patient	Response on a scale (7-point Likert scale)	Mean change	x	x	x
11	Guzik et al., [49]	2019	1	Patients’ perception of change in gait	Patient	Positive change vs. no change vs. worse	Mean change, regression analysis, ROC	1	SEM	x
12	Wu et al., [50]	2019	1	Perceived recovery score of the SIS 3.0	Patient	10–15% change	Mean change	1	0.5 SD	x
13	Fulk et al., [51]	2018	2	mRS, SIS	Patient, clinician	Improvement in mRS ≥ 1; increase in SIS by 10%	ROC	x	x	x
14	Chen et al., [52]	2018	3	Motor Activity Log, Stroke Impact Scale, and Nottingham Extended Activities of Daily Living	Patient, clinician	10–20% increase	Mean change	1	0.5 SD	x
15	Song et al., [53]	2018	1	GROC	Patient	Response on a scale (15-point GROC (global rating of change) scale)	ROC	x	x	x
16	Cranston et al., [54]	2017	x	x	x	x	x	x	x	1 points; 1.1–1.5%
17	New et al., [55]	2017	1	Global rating of change	Patient, clinician	Response on a scale (7-point Likert scale)	Mean change	1	Effect size	x
18	Lundquist et al., [56]	2017	1	Global rating of change	Patient	Response on a scale (7-point Likert scale)	ROC	x	x	x
19	Fulk et al., [57]	2017	1	Global rating of change	Patient, clinician	Response on a scale (5-point Likert scale)	ROC	1	SEM	x
20	Correa et al., [58]	2017	x	x	x	x	x	1	SEM	x
21	Pandian et al., [59]	2016	2	Global rating of patient-perceived changes (GRPPC); Functional Ambulation Classification (FAC)	Patient	Improvement in score ≤2 on GRPPC or ≥1 on FAC	ROC	x	x	x
22	Chen et al., [60]	2016	1	SIS	Patient	10–15% change	Mean change	1	0.5 SD	x
23	Kim et al., [61]	2015	2	mRS, Barthel Index (BI)	Patient	mRS: response on a 5-point Likert scale; BI: difference of at least 4 points	Mean change	x	x	x
24	Bohannon et al., [62]	2013	1	Decrease in assistance required	Patient	Decrease or not	ROC	x	x	x
25	Page et al., [63]	2012	1	Global rating of change	Clinician	Response on a scale (5-point Likert scale)	ROC	x	x	x
26	Arya et al., [64]	2011	2	mRS, GRPPC	Patient, clinician	mRS ≥ 1; GRPPC ≥ 2	ROC	x	x	x
27	Wu et al., [65]	2011	1	SIS	Patient	5 to 7.5 points (10–15% of the total scale score range) on the ADL/IADL domain of the SIS	Mean change	1	0.2 SD	x
28	Wang et al., [66]	2011	1	SIS	Patient	10–15% change	Mean change	1	0.2 SD	x
29	Fulk et al., [67]	2011	2	GROC (patient and clinician)	Patient, clinician	Response on a scale (15-point Likert scale)	ROC	x	x	x
30	Lin et al., [68]	2011	1	SIS	Patient	10–15% change	Mean change	1	0.5 SD	x
31	Fulk et al., [69]	2010	2	GROC (patient and clinician)	Patient, clinician	Response on a scale (15-point Likert scale)	ROC	x	x	x
32	Tilson et al., [70]	2010	1	mRS	Clinician	Improvement in mRS ≥ 1	ROC, regression analysis	x	x	x
33	Hsieh et al., [71]	2008	1	GROC	Patient	Response on a scale (15-point Likert scale)	Mean change	x	x	x
34	Lang et al., [72]	2008	1	Global rating of patient-perceived changes in their affected upper extremity	Patient	Response on a scale (7-point Likert scale)	Mean change	x	x	x
35	Hsieh et al., [73]	2007	1	Global ratings of ADL function	Patient	Response on a scale (15-point Likert scale)	Mean change	1	SEM	x
36	Beninato et al., [74]	2006	1	Global rate of overall clinical change in function	Clinician	Response on a scale (15-point Likert scale)	ROC	x	x	x

**Table 3:** Interpretation of already published trials with regards to MCID thresholds found in this review using the same scales.

Sl No.	Scale	Studies Reporting MCID Thresholds and Included in This Review	MCID Thresholds Reported	Example of Study Evaluating the Same Scale in Stroke Patients	Title of Study	Change in Score Considered Significant	Interpretation as Per Reported MCID	Interpretation of Clinical and Statistical Relevance
1	Berg Balance Scale (BBS)	Tamura et al., 2021 [39]	4 to 5 points	Marques-Sule et al., 2021 [91]	Effectiveness of Nintendo Wii and Physical Therapy in Functionality, Balance, and Daily Activities in Chronic Stroke Patients	Mean SBS difference between VRWiiG group (intervention) and CPTG (conventional physical therapy group) = 6.4 (95% CI 0.2 to 12.6)	Results achieved MCID threshold	Statistically significant and also clinically meaningful 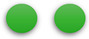
		Song et al. et al., 2018 [53]	12.5 points	-do- [91]	-do-	-do-	Results did not achieve MCID threshold	Statistically significant but not clinically meaningful 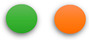
2	MiniBESTest	Beauchamp et al., 2021 [40]	4 points	No suitable study found for comparison	-	-	-	-
3	Five-Repetition Sit-to-Stand test (5STS) in seconds	Augustin et al., 2021 [41]	0.76 to 1.18 s: all patients	No suitable study found for comparison	-	-	-	-
4	Knee range of motion (ROM)	Guzik et al., 2020 [43]	8.48 degrees: affected side	No suitable study found for comparison	-	-	-	-
5	Gait speed	Agustin et al., 2021 [41]	0.09 m/s: all patients	Kim et al., 2021 [92]	Effects of Task-Specific Training after Cognitive Sensorimotor Exercise on Proprioception, Spasticity, and Gait Speed in Stroke Patients: A Randomized Controlled Study	Mean difference between intervention and control group: 0.21 m/s	Results achieved MCID threshold	Statistically significant and also clinically meaningful 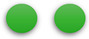
		Bohannon et al., 2013 [62]	0.13 m/s	-do- [92]	-do-	-do-	Results achieved MCID threshold	Statistically significant and also clinically meaningful 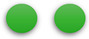
		Fulk et al., 2011 [67]	0.17 m/s	-do- [92]	-do-	-do-	Results achieved MCID threshold	Statistically significant and also clinically meaningful 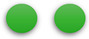
		Tilson et al., 2010 [70]	0.16 m/s	-do- [92]	-do-	-do-	Results achieved MCID threshold	Statistically significant and also clinically meaningful 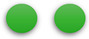
6	Sitting Balance Scale (SBS)	Alzyoud et al., 2020 [44]	5 points	Lee et al., 2021 [93]	The relationship between sitting balance, trunk control and mobility with predictive for current mobility level in survivors of subacute stroke	Cutoff score for SBS using ROC was calculated as 28.5 points for predicting mobility of subacute stroke survivors	Results achieved MCID threshold	Statistically significant and also clinically meaningful 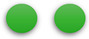
7	Function in Sitting Test (FIST)	Alzyoud et al., 2020 [44]	4 points	No suitable study found for comparison	-	-	-	
8	Dysphagia Severity Rating Scale (DSRS)	Everton et al., 2020 [45]	1 to 2.5 points	Bath et al., 2020 * [94]	Pharyngeal electrical stimulation for neurogenic dysphagia following stroke, traumatic brain injury or other causes: Main results from the PHADER cohort study	DSRS improved significantly in all; dysphagia: 6.5 to 6.7 points	Results achieved MCID threshold	Statistically significant and also clinically meaningful 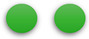
9	Substantial reperfusion (TICI 2b-3)	Lin et al., 2020 [46]	3.1–5%	Nogueira et al., 2018 [95]	Safety and Efficacy of a 3-Dimensional Stent Retriever With Aspiration-Based Thrombectomy vs. Aspiration-Based Thrombectomy Alone in Acute Ischemic Stroke Intervention: A Randomized Clinical Trial	15%	Results achieved MCID threshold	Statistically significant and also clinically meaningful 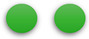
10	Modified Ashworth Scale (MAS)	Chen et al., 2019 [47]	UE: 0.48, LE: 0.45 points	de Gooijer-van de et al., 2016 [96]	Estimation of tissue stiffness, reflex activity, optimal muscle length and slack length in stroke patients using an electromyography driven antagonistic wrist model	1 point	Results achieved MCID threshold	Statistically significant and also clinically meaningful 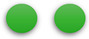
11	Fugl-Meyer assessment of the upper extremity (FMA-UE)	Hiragami et al., 2019 [48]	UE: 12.9 points	Wen et al., 2022 [97]	Therapeutic Role of Additional Mirror Therapy on the Recovery of Upper Extremity Motor Function after Stroke: A Single-Blind, Randomized Controlled Trial	Mean difference between intervention and control group: 6.32 points	Results did not achieve MCID threshold	Statistically significant but not clinically meaningful 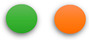
		Lundquist et al., 2017 [56]	≥4 points	-do- [97]	-do-	-do-	Results achieved MCID threshold	Statistically significant and also clinically meaningful 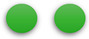
		Arya et al., 2011 [64]	10 points	-do- [97]	-do-	-do-	Results did not achieve MCID threshold	Statistically significant but not clinically meaningful 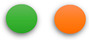
		Page et al., 2012 [63]	4.25 to 7.25 points	-do- [97]	-do-	-do-	Results achieved MCID threshold	Statistically significant and also clinically meaningful 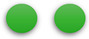
12	Fugl-Meyer assessment of the lower extremity (FMA-LE)	Pandian et al., 2016 [59]	6 points	Kwong et al., 2019 * [98]	Cutoff Score of the Lower-Extremity Motor Subscale of Fugl-Meyer Assessment in Chronic Stroke Survivors: A Cross-Sectional Study	≥21 points	Results achieved MCID threshold	Statistically significant and also clinically meaningful 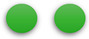
13	Wisconsin Gait Scale (WGS)	Guzik et al., 2019 [49]	2.5 points	Pizzi et al., 2007 [99]	Gait In Hemiplegia: Evaluation Of Clinical Features With The Wisconsin Gait Scale	1.5 points	Results did not achieve MCID threshold	Statistically significant but not clinically meaningful 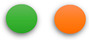
14	Montreal Cognitive Assessment (MoCA)	Wu et al., 2019 [50]	1.22 to 2.15 points	No suitable study found for comparison	-	-	-	-
15	6-min walk test (6MWT)	Fulk et al., 2018 [51]	71 m	Busk et al., 2022 * [100]	Inter-rater reliability and agreement of 6 Minute Walk Test and 10 Meter Walk Test at comfortable walk speed in patients with acute stroke.	75.4 m	Results achieved MCID threshold	Statistically significant and also clinically meaningful 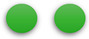
16	Arm Accelerometer	Chen et al., 2018 [52]	547–751 counts	No suitable study found for comparison	-	-	-	
17	Modified Rankin Scale (mRS)	Cranston et al., 2017 [54]	1 level change	Berkhemer et al., 2015 [101]	A Randomized Trial of Intraarterial Treatment for Acute Ischemic Stroke (MR CLEAN trial)	1 point	Results achieved MCID threshold	Statistically significant and also clinically meaningful 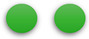
		-do- [54]	-do-	Campbell et al., 2017 [102]	Tenecteplase versus alteplase before endovascular thrombectomy (EXTEND-IA TNK): A multicenter, randomized, controlled study	1 point	Results achieved MCID threshold	Statistically significant and also clinically meaningful 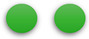
18	Safe Acute Ischaemic stroke treatment	Cranston et al., 2017 [54]	1.1–1.5%	Samsa et al., 2001 * [103]	Have Randomized Controlled Trials of Neuroprotective Drugs Been Underpowered?	2 to 3%	Results achieved MCID threshold	Statistically significant and also clinically meaningful 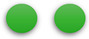
19	de Morton Mobility Index (DEMMI)	New et al., 2017 [55]	2.9 to 8 points	Braun et al., 2021 * [104]	A generic outcome assessment of mobility capacity in neurorehabilitation: measurement properties of the de Morton Mobility Index	15 points	Results achieved MCID threshold	Statistically significant and also clinically meaningful 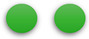
20	Arm Motor Ability Test (AMAT)	Fulk et al., 2017 [57]	≥0.44 points	Kunkel et al., 1999 * [105]	Constraint-Induced Movement Therapy for Motor Recovery in Chronic Stroke Patients	2.04 points	Results achieved MCID threshold	Statistically significant and also clinically meaningful 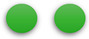
21	Gait Deviation Index (GDI)	Correa et al., 2017 [58]	9.4 points: non-paretic limb, 7.5 points: paretic limb	No suitable study found for comparison	-	-	-	-
22	EuroQoL 5-Dimensions Questionnaire (EQ-5D-5L); Visual analog scale (EQ-VAS)	Chen et al., 2016 [60]	EQ-Index: 0.1 points, EQ-VAS: 8.61–10.82 points	Golicki et al., 2015 * [106]	Comparing responsiveness of the EQ-5D-5L, EQ-5D-3L and EQ VAS in stroke patients	EQ-5D-5L: 0.11 points, EQ-VAS: 6.4 points	EQ-5D-5L results achieved MCID threshold but EQ-VAS results did not achieve MCID threshold	EQ-5D-5L: statistically significant and also clinically meaningful 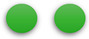 EQ-VAS: statistically significant but not clinically meaningful 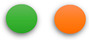
		Kim et al., 2015 [61]	EQ-5D: 0.08–0.12	No suitable study found for comparison	-	-	-	-
23	Nottingham Extended Activities of Daily Living (NEADL) scale	Wu et al., 2011 [65]	6.1 points	Logan et al., 1997 [107]	A randomized controlled trial of enhanced Social Service occupational therapy for stroke patients	At 3 months: intervention vs. control group = 5 points At 6 months: intervention vs. control group = 2 points	Results did not achieve MCID threshold	Statistically significant but not clinically meaningful 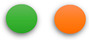
24	ABILHAND questionnaire	Wang et al., 2011 [66]	0.26 to 0.35 points	Ekstrand et al., 2023 * [108]	Clinical interpretation and cutoff scores for manual ability measured by the ABILHAND questionnaire in people with stroke	1.78 points	Results achieved MCID threshold	Statistically significant and also clinically meaningful 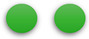
25	Stroke-Specific Quality of Life Scale (SS-QOL)	Lin et al., 2011 [68]	Mobility: 1.5–2.4, self-care: 1.2–1.9, UE function: 1.2–1.8	No suitable study found for comparison	-	-	-	-
26	Stroke Impact Scale-16 (SIS-16)	Fulk et al., 2010 [69]	9.4–14.1 points	Wu et al., 2012 * [109]	Effect of Therapist-Based Versus Robot-Assisted Bilateral Arm Training on Motor Control, Functional Performance, and Quality of Life After Chronic Stroke: A Clinical Trial	Change pre–post intervention: RBAT: 5.3 points, TBAT: 3.4 points	Results did not achieve MCID threshold	Statistically significant but not clinically meaningful 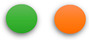
27	Stroke Rehabilitation Assessment of Movement (STREAM) measure	Hsieh et al., 2008 [71]	UE: 2.2, LE: 1.9, mobility: 4.8 points	Ahmed et al., 2003 * [110]	The Stroke Rehabilitation Assessment of Movement (STREAM): A Comparison With Other Measures Used to Evaluate Effects of Stroke and Rehabilitation	UE:15, LE: 15, mobility: 17 points	Results achieved MCID threshold	Statistically significant and also clinically meaningful 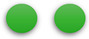
28	Grip Strength	Lang et al., 2008 [72]	5.0 kg	Sunderland et al., 1989 * [111]	Arm function after stroke. An evaluation of grip strength as a measure of recovery and a prognostic indicator	Pre–post intervention change: 1 kg	Results did not achieve MCID threshold	Statistically significant but not clinically meaningful 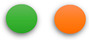
29	Action Research Arm Test (ARAT)	Lang et al., 2008 [72]	12 points	Rabadi et al., 2006 * [112]	Comparison of the action research arm test and the Fugl-Meyer assessment as measures of upper-extremity motor weakness after stroke	13 points	Results achieved MCID threshold	Statistically significant and also clinically meaningful 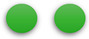
		-do- [72]	-do-	Page et al., 2004 [113]	Efficacy of modified constraint-induced movement therapy in chronic stroke: a single-blinded randomized controlled trial	11.4 points	Results did not achieve MCID threshold	Statistically significant but not clinically meaningful 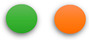
30	Wolf Motor Function Test (WMFT)	Lang et al., 2008 [72]	1.0 points	Lindenberg et al., 2010 [114]	Bihemispheric brain stimulation facilitates motor recovery in chronic stroke patients	Mean difference between intervention and control group: 0.5 points	Results did not achieve MCID threshold	Statistically significant but not clinically meaningful 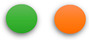
31	Motor Activity Log (MAL)	Lang et al., 2008 [72]	1.0 points	Hammer et al., 2010 * [115]	Responsiveness and validity of the Motor Activity Log in patients during the subacute phase after stroke	1.0 points	Results achieved MCID threshold	Statistically significant and also clinically meaningful 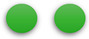
32	Barthel Index (BI)	Hsieh et al., 2007 [73]	1.85 points	Nasb et al., 2019 * [116]	Comparison of the effects of modified constraint-induced movement therapy and intensive conventional therapy with a botulinum-a toxin injection on upper limb motor function recovery in patients with stroke	Change between groups (BTX-mCIMT vs. BTX-ICT groups) at 4 weeks: 9.6 points	Results achieved MCID threshold	Statistically significant and also clinically meaningful 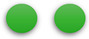
33	FIM instrument	Beninato et al., 2006 [74]	Total: 22, motor: 17, cognitive: 3	Putten et al., 1999 * [117]	Measuring change in disability after inpatient rehabilitation: comparison of the responsiveness of the Barthel Index and the Functional Independence Measure	Pre–post intervention mean change in score: FIM total 21.9 ± 19.0; FIM motor 19.1 ± 16.1; FIM cognitive 2.8 ± 4.8	FIM motor achieved MCID threshold FIM total and FIM cognition did not achieve MCID threshold	FIM motor: statistically significant and also clinically meaningful 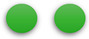 FIM total and FIM cognition: statistically significant but not clinically meaningful 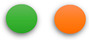
34	Physical Component Summary (PCS) score of the Short Form 36 (SF-36)	Fu et al., 2021 [42]	1.8 to 3.0 units	Laffont et al., 2020 * [118]	Rehabilitation of the upper arm early after stroke: Video games versus conventional rehabilitation. A randomized controlled trial	Baseline to 6 month mean change in intervention group: 2.8 ± 6.5 points	Results achieved MCID threshold	Statistically significant and also clinically meaningful 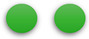

* Trials in which a comparison of the scale was conducted pre and post intervention in the same group and not with controls. BTX-mCIMT—botulinum-A toxin (BTX) injection with modified constraint-induced movement therapy, BTX-ICT—BTX with high-dose conventional therapy, RBAT—robot-assisted bilateral arm training, TBAT—therapist-based bilateral arm training. First Green circle—Statistically significant; Second Green circle—Clinically meaningful also; Orange circle—Not clinically meaningful.

## Data Availability

The authors confirm that the data supporting the findings of this study are available within the article and its Supplementary Materials. Any further data needed can be made available from the corresponding author, V.Y.V., upon reasonable request.

## Supplementary Materials

The following supporting information can be downloaded at: https://www.mdpi.com/article/10.3390/brainsci14010080/s1, reference [39,40,41,42,43,44,45,47,49,50,51,52,53,54,55,57,58,60,61,65,66,68,69,71,72,74,133,134,135,136,137,138,139,140,141,142,143,144,145,146,147,148,149,150,151,152,153,154,155,156,157,158,159,160,161,162,163,164,165,166,167,168,169,170,171,172,173,174,175,176,177,178,179,180,181,182,183,184,185,186,187,188] are cited in supplementary materials. Details about the MCIDs of various other scales, like the Mini-Balance Evaluation Systems Test (Mini-BESTest), Five-Repetition Sit-to-Stand Test (5STS), Knee Range of Motion (ROM), Wisconsin Gait Scale (WGS), Arm Motor Ability Test (AMAT), Stroke Rehabilitation Assessment of Movement (STREAM), Stroke Impact Scale-16 (SIS-16), and Nottingham Extended Activities of Daily Living (NEADL) scale.

## Abbreviations

10mWT	10-Meter Walk Test
5STS	Five-Repetition Sit-to-Stand Test
ADLs	Activities of Daily Living
AMAT	Arm Motor Ability Test
BBS	Berg Balance Scale
BI	Barthel Index
BMI	Body Mass Index
CGIC	Clinician Global Impression of Change
CGS	Comfortable Gait Speed
EBM	Evidence-Based Medicine
EVT	Endovascular Therapy
FAC	Functional Ambulation Categories
FIST	Function in Sitting Test
FMA-LE	Fugl-Meyer Assessment of the Lower Extremity
FMA-UE	Fugl-Meyer Assessment of the Upper Extremity
GROC	Global Rating of Change
GRPPC	Global Rating of Patient-Perceived Changes
HRQoL	Health-Related Quality of Life
MCID	Minimal Clinically Important Difference
MDC	Minimum Detectable Change
Mini-BESTest	Mini-Balance Evaluation Systems Test
mRS	Modified Rankin Scale
NEADL	Nottingham Extended Activities of Daily Living
PROM	Patient-Reported Outcome Measure
PRO	Patient-Reported Outcome
PROMs	Patient-Reported Outcome Measures
ROC	Receiver Operating Characteristic
SD	Standard Deviation
SEM	Standard Error of Measurement
SIS	Stroke Impact Scale
SIS-16	Stroke Impact Scale-16
STREAM	Stroke Rehabilitation Assessment of Movement
TICI	Thrombolysis in Cerebral Infarction
WGS	Wisconsin Gait Scale
