# Supplementary material for: Minimal Clinically Important Difference of Scales Reported in Stroke Trials: A Review
Source: Brain Sci. 2024 Jan 13;14(1):80. doi: 10.3390/brainsci14010080 (PMC10813687; doi:10.3390/brainsci14010080)
Supplement: Supplementary file 1 [file brainsci-14-00080-s001.zip › brainsci-2802059-supplementary.pdf]

Supplementary Material

Details about the MCIDs of various other scales, like the Mini-Balance Evaluation Systems Test (Mini-BESTest), Five-Repetition Sit-to-Stand test (5STS), Knee Range of Motion (ROM), Wisconsin Gait Scale (WGS), Arm Motor Ability Test (AMAT), Stroke Rehabilitation Assessment of Movement (STREAM), Stroke Impact Scale-16 (SIS-16), Nottingham Extended Activities of Daily Living (NEADL) scale, and a few others, are provided in the supplementary material.

Supplementary Material: Additional MCID measures reported in stroke patients in the literature under the section Rehabilitation.

1. **Berg Balance Scale (BBS)**

The BBS consists of 14 items scored on a five-point ordinal scale, ranging from 0 to 4 (0 indicates lowest level of function; 4 indicates highest level of function), with a maximum total score of 56. The Berg Balance Scale is a clinician-reported scale. The 14 items of BBS include the following: 1. Standing unsupported, 2. Sitting unsupported, 3. Standing to sitting, 4. Transfers, 5. Standing with eyes closed, 6. Standing with feet together, 7. Reaching forward with outstretched arm, 8. Retrieving object from floor, 9. Turning to look behind, 10. Turning 360 degrees, 11. Placing alternate foot on stool, 12. Standing with one foot in front, 13. Standing on one foot, and 14. Tandem stance. Participants presenting a score of 41 to 56 points are described as "independent"; scores of 21 to 40 are interpreted as "walking with assistance"; and scores of 0 to 20 are generally classified as "wheelchair-bound" [1–3]. Two studies have evaluated the MCID for the BBS in stroke patients using anchor-based methods.

Tamura et al., 2021, estimated the MCID of the BBS for 80 patients with early subacute stroke (mean duration since stroke onset: 31.3 ±13.0 days) who needed walking assistance and those who did not. The Functional Ambulation Categories (FACs) were used as anchors to estimate the MCID. The estimated MCID for the assisted walking group was five points, with good discrimination accuracy, while the corresponding value for the unassisted walking group was four points, with lower discrimination accuracy [4].

Song et al., 2018, estimated the MCID of the BBS in 73 participants with acute stroke who received rehabilitation in the form of physical therapy for 4 weeks. The anchor used in their study was a self-rating global rating of change. The MCID for BBS score change was 12.5 points for both males and females [5].

2. **Mini-Balance Evaluation Systems Test (Mini-BESTest)**

The Mini-BESTest is a 14-item test scored on a three-level ordinal scale. It assesses dynamic balance, a unidimensional construct, and includes 14 items addressing four of the six sections of the original BESTest. The Mini BESTest is a clinician-reported scale. The test is divided into four subcomponents (anticipatory postural adjustments, postural responses, sensory orientation, and dynamic gait) and scored on a three-level ordinal scale with a total score out of 28 points (higher scores indicate better balance) [6–8].

Beauchamp et al., 2021, assessed the MCID of the Mini-BESTest in 50 individuals post stroke (mean duration since stroke onset: 124.4±106.5 days) before and after conventional rehabilitation (mean treatment duration: 121.8±74.3 days). A combination of anchor (global rating of change in balance scale as the anchor) and distribution (SEM)-based approaches were used to determine the MCID. The MCID for detecting small changes was determined to be four points and that for detecting substantial changes was five points [9].

3. **Sitting Balance Scale (SBS) and Function in Sitting Test (FIST)**

The Sitting Balance Scale (SBS) consists of 11 items that represent different functional abilities of sitting balance, such as turning, reaching, and picking up objects. Each item is scored on a five-point ordinal scale with a maximum score of 44, rated on a 0 to 4 Likert scale. The SBS has been validated in individuals with stroke [10,11]. The 11 items of the SBS are clinician-assessed and read as

follows: 1. Reaching forward with outstretched arm while sitting, 2. Reaching laterally while sitting, 3. Picking up objects from the floor while sitting unsupported on foam, 4. Turning to look behind over left and right shoulders when sitting, 5. Sitting unsupported with eyes closed, 6. Sitting unsupported with feet together, 7. Sitting unsupported with feet apart, 8. Sitting unsupported with arms crossed, 9. Sitting unsupported with arms folded, 10. Sitting unsupported with hands on thighs, and 11. Sitting unsupported with hands on knees.

The FIST is a clinical assessment tool used to evaluate functional abilities while sitting. The test consists of 14 items. Each item is scored on a 0 to 4 ordinal scale (based on the assistance needed) and involves measuring the patient's ability to perform functional activities such as reaching, twisting, and lifting while sitting in a chair [12]. The 14 items of the FIST are measured by a clinician and read as follows: 1. Static sitting, 2. Sitting, shake "no" left and right, 3. Sitting, eyes closed, 4. Sitting, lift foot: dominant side, lift foot 1 inch twice, 5. Pick up object from behind: object at midline, hand breadth posterior, 6. Forward reach: use dominant arm, must complete full motion, 7. Lateral reach: use dominant arm, must complete full motion, 8. Sit to stand, 9. Stand to sit, 10. Sit with feet on stool, 11. Sit with feet on stool and eyes closed, 12. Sit with feet on stool and head turns left and right, 13. Sit with feet on stool and arms crossed over chest, 14. Sit with feet on stool and arms crossed over chest and eyes closed [12].

Alzyoud et al., 2021, used both anchor- and distribution-based methods to assess the MCID for the SBS and FIST using the Barthel Index (BI) as an anchor. Forty-three patients with a mean stroke duration of 106±247.7 days received physical therapy for a mean duration of 23.8±12.8 days. The MCID for the SBS and FIST was estimated at 4.5 points and 3.5 points, respectively, using the anchor-based method; and at 3.5 points and 4.8 points, respectively, using the distribution-based method. Overall, a change of five points in the SBS and four points in the FIST were considered to indicate a meaningful improvement in sitting balance [13].

4. **Five-Repetition Sit-to-Stand Test (5STS)**

The 5STS (Five Times Sit-to-Stand) test is a clinical assessment tool used to evaluate functional mobility using a chair without an armrest and with a specific height (43 cm) and depth (47.5cm). The test involves completing five repetitions of the sit-to-stand task, with timing beginning when the patient's back leaves the backrest and stopping once the back touches the backrest for the fifth time. Participants who are unable to complete the task within 1 minute are given a score of 60 seconds. The normal score for the Five Times Sit-to-Stand Test (5STS) varies by age group and gender. ranging from 6.0±1.4 sec at 20-29 years to 16.7±4.5 sec (males) and 17.2±5.5 sec (females) at 80-89 years [14–16].

Martín-San Agustín et al., 2021, evaluated the MCID for the 5STS for community ambulation in 111 poststroke patients at various stages of rehabilitation and severity levels (mean duration since stroke onset: 51.8 ± 31.5). The MCID was calculated with two anchor-based methods. The 5STS was evaluated along with comparator instruments (gait speed and functional ambulatory category) at baseline, 4 weeks (Stage 1), and 8 weeks (Stage 2), along with a global rating of change (GROC) change as an external criterion. The MCID for limited community ambulators was almost similar at both time points, i.e., around 3 s, while for household ambulators, the MCID was 1.9 seconds (s) at 4 weeks and 0.72 s at 8 weeks [17].

5. **Knee Range of Motion (ROM)**

Impaired knee ROM following a stroke can lead to difficulties with activities of daily living, such as walking and climbing stairs. Knee range of motion refers to the degree of movement that occurs at the knee joint in flexion and extension. It is an important measure of knee function and is used to assess joint mobility and flexibility [18,19].

Guzik et al., 2020, estimated the MCID for knee range of motion (ROM) in the sagittal plane for the affected and unaffected side in poststroke patients. Fifty patients at mean poststroke duration of 42 (8-120) days were included in the study. The study used a six-camera motion capture system and force plates

to collect kinematic knee data while subjects walked 10 meters at their natural pace without shoes. Passive markers were placed on specific points on the body, and a 3D skeletal model was created for each subject. The data were processed with software, and the complete range of knee flexion and extension during a gait cycle was analyzed for both the affected and unaffected sides. The study included a minimum of six gait cycles for each subject, and mean values of biomechanical gait parameters were calculated for both the affected and unaffected sides using four different methods ((anchor-based methods (using patients◎perceptions of the knee sagittal ROM as an anchor) and distribution-based methods (SEM)). The MCID for the knee sagittal ROM on the affected side was estimated at 8.48°, whereas for the unaffected side, the value was 6.81°[20].

6. **Wisconsin Gait Scale (WGS)**

The Wisconsin Gait Scale (WGS) is a comprehensive tool for analyzing gait that takes into account 14 observable gait parameters which are divided into four subscales based on the different phases of gait. The first subscale, related to the stance phase of the affected leg, includes five items such as the use of a hand-held gait aid and stance time on the impaired side. The second subscale, related to the toe-off phase of the affected leg, includes two items, namely guardedness and hip extension of the affected leg. The third subscale, related to the swing phase of the affected leg, includes six items such as external rotation during initial swing and toe clearance. The last subscale, related to the heel strike of the affected leg, includes one item: initial foot contact of the affected leg. A summary score is calculated based on the 14 items, ranging from 13.35 to 42 points. Items 2-10 and 12-14 are summed, and items 1 and 11 both contribute to the summary score but are weighted differently before being added to the total score. A higher WGS score indicates poorer gait performance and greater gait deviations [21].

Guzik et al., 2019, evaluated the MCID in 50 stroke patients in the chronic recovery phase at a mean of 42 [8–120] days from stroke onset. The patients followed their usual daily rehabilitation regimen. Gait analysis was conducted using two synchronized video cameras positioned to record images in both the frontal and sagittal planes. The cameras were set up to visualize three walking trials for both the affected and unaffected sides, totaling six ambulation trials. The walking distance was 10 m, and the cameras were placed in such a way that one camera recorded the gait in the frontal plane while the other camera captured the image in the sagittal plane. The subjects were instructed to walk at a self-selected (comfortable) speed using their regular orthopedic aids. Finally, a senior gait analysis physiotherapist interpreted the recordings and evaluated them using the Wisconsin Gait Scale. The MCID was assessed at 4 weeks of study inclusion using anchor (patient-reported change as the anchor) and distribution (SEM)-based methods and estimated using linear regression analysis and specification of ROC curve. Based on the largest estimate, the MCID for the WGS was estimated at 2.25 points, meaning that an increase in the WGS score by 2.5 points is considered to indicate a meaningful improvement in gait function [22].

7. **Dysphagia Severity Rating Scale (DSRS)**

The DSRS (Dysphagia Severity Rating Scale) is a 16-item scale used by clinicians to assess the severity of dysphagia in patients. It is based on the Dysphagia Outcome and Severity Scale (DOSS) (O◎Neil) and evaluates how much modification is required for safe oral intake, as well as the level of supervision needed. The DSRS has three subscales that assess fluid and dietary intake as well as supervision and the scores from these subscales are totaled to give an overall score ranging from 0 (indicating the least severe dysphagia) to 12 (indicating the most severe dysphagia). Each subscale has a five-level ordinal assessment that ranges from normal (score 0) to no intake (score 4) [23].

Everton et al., 2020, evaluated the MCID for the Dysphagia Severity Rating Scale (DSRS) for poststroke dysphagia using anchor-based, distribution-based, and survey methods using data from one previous RCT (STEPS) (Bath) and a survey. The survey was conducted on 84 UK-based speech and language therapists (SLTs). The majority of the SLTs had more than 10 years of

experience. The MCIDs for the DSRS using anchors were as follows: aspiration at week 2 and oral vs non-oral feeding at week 2: 2.5 points and 1.0 points, respectively. Using 0.5 SD and SEM, the MCIDs for the DSRS were as follows: 1.9 and 0.3 points, respectively. Using the survey method, the MCID was one point [24].

8. **Arm Motor Ability Test (AMAT)**

The Arm Motor Ability Test (AMAT) is utilized to assess upper extremity functional limitation in rehabilitation trials for individuals who have suffered a stroke, among other neurological and musculoskeletal conditions. The AMAT is composed of 13 bimanual and unimanual activities that are part of activities of daily living (ADLs), such as cutting meat, combing hair, and opening a jar. Each of the 13 activities is further divided into several subtasks, with a total of 28 task components. These task components are evaluated using a Functional Ability Scale that assesses paretic limb use (0 = no use of paretic upper extremity; 5 = comparable to the less affected side) and a Quality of Movement Scale (0 = no movement; 5 = normal movement) [25].

Fulk et al., 2017, assessed the MCID for the AMAT in 146 chronic stroke survivors (mean time since stroke onset = 59.37 ± 63.22 months) with mild to moderate hemiparesis. The MCID was assessed using anchor-based (global rating of change (GROC) scale as the anchor) and distribution-based (SEM) methods at six weeks post intervention (repeated practice of task-specific activities, accompanied by electrical cortical stimulation delivered through a Northstar Stroke Recovery System). A change of 0.44 points or greater in the AMAT was found to be the MCID for functional upper limb movement [26].

9. **Stroke Rehabilitation Assessment of Movement (STREAM) measure**

The STREAM measure is a tool used to assess the movement abilities and basic mobility of stroke patients. It consists of 30 items which are equally divided among three subscales: upper extremity movements, lower extremity movements, and basic mobility. The upper and lower extremity movement items are scored on a three-point scale (0-1-2), while the mobility items are scored on a four-point scale (0-1-2-3). Each limb subscale has a possible score range of 0 to 20 points, while the mobility subscale ranges from 0 to 30 points. The total possible score for the STREAM measure is 70 points [27].

Hseih et al., 2008, assessed the MCID for STREAM in 81 stroke patients (mean time since stroke = 50.1 ± 51.4 days), using an anchor-based method with responses on a scale (15-point Likert scale) as the anchor. The interval between the two assessments for STREAM was 24.2 ±7.1 days. The MCID for STREAM was estimated at 2.2 points for the upper extremity, 1.9 points for the lower extremity, and 4.8 points for overall mobility [28].

10. **Stroke Impact Scale-16 (SIS-16)**

The SIS-16 is a brief version of the Stroke Impact Scale, created through Rasch analysis. It includes 16 items from the physical functioning sections of the original scale, such as strength, hand function, activities of daily living/instrumental activities of daily living, and mobility. Its purpose is to assess the influence of physical functioning on poststroke disability [29,30].

Fulk et al., 2010, estimated the MCID for the SIS-16 in 36 patients at a mean poststroke duration of 58.9 ± 47.1 days who visited outpatient physical therapy (PT) clinics. The mean number of PT sessions attended were 22.9 ± 12.4. The MCID was assessed using anchor-based methods with ROC curves of GROCs reported by physicians and patients as anchors. The MCID for the SIS-16 was estimated to range from 9.4 to 14.1 points depending on the anchor [31].

11. **Nottingham Extended Activities of Daily Living (NEADL) scale**

The NEADL (Nottingham Extended Activities of Daily Living) scale is a tool used to evaluate a patient's level of independence in four areas of daily living, namely kitchen, domestic, mobility, and leisure activities [32], and is widely used in stroke rehabilitation trials [33]. The NEADL scale is based on the World Health Organization's International Classification of Health, Functioning, and Disability model and is used to assess a stroke patient's quality of life. The scale has demonstrated reliability, validity, and responsiveness in stroke patients [34]. It is a patient-reported outcome and each

item on the scale is rated using a four-point scale (0=unable to 3=able), with higher scores representing greater independence.

Wu et al., 2011, estimated the MCID of NEADL in 78 poststroke patients (mean duration since stroke: 19.7 ± 17.0 months) who received one of four rehabilitation interventions. A mean change in NEADL score of 6.1 points was considered clinically important [35].

12. **Arm accelerometry**

An accelerometer is a device that measures acceleration. It can be used to measure the gross amount of arm movement for upper extremity rehabilitation [36,37].

Chen et al., 2018, assessed the MCID for arm accelerometry on 82 patients with chronic stroke. To measure the real-world movement of the arm, they used an arm accelerometer called the MicroMini-Motionlogger activity monitor (Ambulatory Monitoring, NY, USA). The monitor recorded the acceleration of the arm at a rate of 10 Hz and summed it over a 1-minute period. The accelerometer data were then processed using ACT-Millennium software and Action4 software. Mean counts of the affected side were used to represent the participants' affected arm activity in their natural living environment. To avoid bias, accelerometers were worn on both wrists continuously for three days before and after the intervention, except when bathing. Participants were also given daily monitoring logs to document their routines throughout the day and were educated on the proper use of the monitors. Therapists collected the daily logs after participants completed the evaluation to ensure the proper use of the accelerometers by patients.

Both anchor (Motor Activity Log (MAL), Stroke Impact Scale (SIS), and Nottingham Extended Activities of Daily Living (NEADL) as anchors) and distribution (0.5 SD)-based methods were used. While the MAL and SIS are patient-reported and use a Likert scale, the NEADL is a clinician-reported scale. The MCID range for the arm accelerometer was estimated at 547–751 mean counts [38].

13. de Morton Mobility Index (DEMMI)

The DEMMI is a 15-item scale that can be used to evaluate functional mobility in patients with stroke [39]. The DEMMI is administered by a clinician following the direct observation of the patient's performance of fifteen hierarchical mobility tasks, of which three require a seated position, three involve lying down, four focus on maintaining a stable posture, two involve walking, and three require performing movements while maintaining balance. These tasks are scored on a scale of 0 to 2, with eleven items having a score range of 0 to 1 and the remaining four items having a score range of 0 to 2.

New et al., 2017, assessed the MCID for the DEMMI on 366 poststroke (mean duration not mentioned) patients who underwent rehabilitation for a median of 25 days (IQR 15–48). Both anchor-based (seven-point Likert assessment of global change in mobility as anchor) and distribution-based (0.5 SD, 0.8 SD) methods were used to assess the MCID. In addition to the patient, the treating physical therapist and the patient's treating rehabilitation physician also had their global rating of the patient's mobility change recorded, both blinded to each other's and the patient's report. The MCID for the DEMMI was estimated at 8.0 points using the anchor-based method and 2.9 points using the distribution-based method [40].

14. **Gait Deviation Index (GDI)**

The GDI is a clinical assessment tool used to evaluate gait function in individuals with conditions that affect motor control, including stroke. The test measures the deviation of a patient's gait pattern from normal gait, providing a quantitative measurement of gait impairments [41].

Correa et al., 2017, assessed the MCID for the GDI on 20 poststroke patients at a mean duration from stroke onset at 6.2 ± 3.2 months who were admitted to a rehabilitation center. All study participants were evaluated for their gait performance in the hospital's gait laboratory during two sessions (test and re-test) with a time interval of 2 to 7 days. The Helen Hayes marker set was used to place reflective markers on the participants' skin during both sessions. The participants were instructed to walk barefoot on a 10-meter path at a self-

selected pace, performing at least six trials. Kinematic data were captured at 5 meters, in the middle of the path, using a motion capture system equipped with six infrared cameras. The data were sampled at 60 Hz and low-pass filtered at 6 Hz with a fourth-order digital Butterworth filter. To calculate the degree of gait pathology, GDI values were determined using an Excel spreadsheet developed by Schwartz and Rozumalski (Schwartz). The distance between an individual's gait vectors and the average vector obtained from a group of healthy subjects, termed the normal group, was calculated to assess the extent of gait pathology. The GDI was assessed at a mean interval of 5.4 ± 2.1 days. The MCID of the GDI was estimated at 9.4 points for the non-paretic limb and 7.5 points for the paretic limb [42].

15. **Grip strength, composite upper extremity strength, Action Research Arm Test (ARAT), Wolf Motor Function Test (WMFT), Motor Activity Log (MAL), and duration of upper extremity use**

Lang et al., 2008, estimated the MCID of various scales that can be applied to stroke rehabilitation. Upper extremity function was evaluated using grip strength, composite upper extremity strength, ARAT, the WMFT, MAL, and the duration of arm use measured with accelerometers.

Grip strength during the WMFT (Wolf Motor Function Test) was measured using a dynamometer to determine the maximum amount of force produced during a palmar grip. The Jamar grip dynamometer was used with the handle position set at 3 for all measurements and for all subjects. To assess grip strength, subjects were seated with their upper extremity in 0° of shoulder flexion and 90° of elbow flexion. Three measurements were taken at each session, and the average value was used in the analyses. Grip strength values are expressed in kilograms. Grip strength has been proposed as a surrogate measure for upper extremity outcomes and it is reliably assessed by a variety of stroke professionals. Using a dynamometer to measure grip strength during the WMFT helps to obtain accurate and reliable data on this important aspect of poststroke motor function [43,44].

Another measured used in the study was composite upper extremity strength. The strength of the shoulder, elbow, and wrist flexor and extensors were measured using a hand-held dynamometer following a standard protocol, except that subjects were seated during testing. No warm-up or practice trials were performed prior to testing, and maximal voluntary isometric strength values were recorded in kilograms for each muscle group tested. Subjects who were unable to produce force against the dynamometer were given a score of 0 kg for that particular muscle group. The strength of each muscle group was expressed as the ratio of the affected side's to the unaffected side's maximal isometric force. Ratio values from each muscle group were averaged to form a single, composite score for the affected upper extremity for each subject [45,46].

The Action Research Arm Test (ARAT) is a commonly used assessment tool to evaluate activity limitations of the upper extremity. It is composed of 19 items that assess different upper extremity movements, such as grasp, grip, pinch, and gross movement. The test has high reliability (interrater, .99; test–retest, .98) and validity (Hsieh: ARAT) and has been shown to be strongly correlated with the upper extremity motor portion of the Fugl-Meyer Assessment (STS) and the box and block test. The scores for each item are summed to create subtest and full-scale scores, with a maximum score of 57, indicating normal performance [47,48].

The Wolf Motor Function Test (WMFT) is a standardized assessment tool that is used to evaluate activity limitations of the upper extremity. It consists of 17 items, including 2 strength items and 15 timed task performance items. The test measures simple proximal movements to more complex distal and whole-limb movements. The WMFT provides two scores: (1) a functional ability score that quantifies performance quality and (2) a timed score that quantifies performance speed in seconds. The WMFT has established reliability and validity, and it is a widely used tool in research and clinical practice to assess upper extremity motor function in individuals with stroke and other neurological conditions [49,50].

The Motor Activity Log (MAL) is a measure of self-perceived upper-extremity participation restrictions. It assesses how much and how well patients use their affected arm for activities of daily living (ADLs) over a specified period of time. The MAL uses a semi-structured interview and evaluates 30 specific ADL tasks using a six-point amount of use scale and a six-point quality of movement scale. In one study, the quality of movement scale, or how well the arm functioned in the ADL tasks, was collected at both study day 0 and day 14 assessments and is included in the report. However, the amount of use scale, or how much the arm was used, was not administered at study day 0, so change scores could not be calculated and the results could not be included in the report. Adequate interrater reliability (0.91) and internal consistency (0.81) have been reported for the MAL [51,52].

Assessing the duration of upper extremity use within a 24-hour period can provide a measure of upper extremity participation. In one study, the duration of upper extremity use was measured using uniaxial accelerometers placed on the distal arm just above the wrist. Data were collected in 2-second epochs over a 24-hour period, with accelerometers worn at all times except during personal hygiene. The data from each epoch were used to classify whether the upper extremity was moving or not, and the sum of the epochs when the upper extremity moved was calculated as the duration of upper extremity movement over the 24-hour period. This measure has been shown to be both valid and reliable [53,54].

Lang et al., 2008, evaluated 52 poststroke individuals with hemiparesis to establish the MCIDs of these various scales. The first evaluation was conducted at 9.5 days and the second evaluation at an average of 25.9 days post stroke. An anchor-based method was used, with a global rating of patient-perceived changes in the affected upper extremity as the anchor. The MCIDs estimated for the dominant hand were as follows: grip strength: 5.0 Kg, ARAT: 12 points, WMFT: 1.0 points, MAL score: 1.0 points. For the non-dominant hand, the MCIDs were as follows: grip strength: 6.2 Kg, ARAT: 17 points, WMFT: 1.2 points, MAL: 1.1 points. MCID values could not be determined for composite strength of the dominant hand, WMFT time score of the non-dominant hand, and for the other measures (duration of use) for both affected sides [55].

16. **ABILHAND questionnaire**

The ABILHAND is a Rasch-based self-administered semi-structured item response questionnaire that assesses how difficult patients perceive it to carry out ADLs that involve using both upper limbs bilaterally. The patient is asked to estimate the ease or difficulty of performing each activity when the activities are performed without help, irrespective of the limb(s) the patient actually uses and whatever the strategies used to perform the activity. It utilizes 23 items that evaluate manual ability and it is scored on a three-point response scale [56].

Wang et al., 2011, assessed the MCID for the ABILHAND in 51 patients with stroke (mean duration since stroke onset: 17.57 ±13.43 days) who received upper extremity rehabilitation programs (one of the following three: conventional rehabilitation, unilateral robot-assisted training, bilateral robot-assisted training) for 4 weeks [57]. Both anchor (SIS as the anchor) and distribution (0.2 SD)-based methods were used to estimate the MCID. The MCID for ABILHAND was estimated at 0.26 to 0.35 points.

17. **EuroQoL 5-Dimensions Questionnaire Five-Level version (EQ-5D-5L), Short Form (SF)-36, and Visual Analog Scale (EQ-VAS)**

The EuroQoL 5-Dimensions Questionnaire (EQ-5D) is a reliable and valid measurement tool for health-related quality of life (HRQoL) in various disease populations, including stroke. It consists of five self-reported health state dimensions, namely mobility, self-care, usual activity, pain/discomfort, and depression/anxiety, as well as a visual analog scale (EQ-VAS). The five dimensions' health status can be transformed into a single utility value (EQ-Index score) for economic evaluations of healthcare interventions. The EQ-VAS captures overall subjective health status by using a vertical 0–100-point scale. Compared to the Short-Form 36, the EQ-5D is briefer and simpler, making it easier for stroke patients to complete without missing data. Participants assess their health status using the EQ-5D by selecting one of five severity levels

(ranging from 1, no problem, to 5, unable to/extreme problem) for each of the five dimensions (mobility, self-care, usual activity, pain/discomfort, and depression/anxiety). The participants can also rate their overall health status on the visual analog scale (EQ-VAS). The responses for the five dimensions can be combined to form a five-digit health status profile ranging from '1-1-1-1-1', indicating no problems in any dimension, to '5-5-5-5-5', indicating an extremely poor health status in all five dimensions [58].

The SF-36 v2 (version 2) Health Survey is a patient-reported outcome (PRO) assessment designed to measure the quality of life and functional health and well-being of patients across a variety of health conditions. The SF-36 v2 comprises 36 questions that evaluate multiple dimensions of an individual's functional health and overall well-being. The survey generates an eight-scale profile, which includes bodily pain, physical functioning, physical role, vitality, general health, emotional role, social functioning, and mental health. Furthermore, it provides two summary measures of physical and mental health, along with a preference-based health utility index [59].

Kim et al., 2015, estimated the MCID for the EQ-5D and SF-6D health indices in 484 poststroke patients (mean duration since the first stroke: 9 ± 7.3 years). The study used data from a longitudinal survey administered to stroke patients at baseline and 10 months. No active rehabilitation measures were provided to the patients. The MCIDs for both indices were estimated via an anchor-based method utilizing the mRS and BI as anchors. For the EQ-5D, the MCID estimates ranged from 0.08 to 0.12, while those for the SF-6D ranged from 0.04 to 0.14. The magnitude of MCID values varied between the two utility measures due to the SF-6D index having a broader range than the EQ-5D [60].

Chen et al., 2016, estimated the MCID for the EQ-5D and EQ-VAS in 65 patients with stroke (mean duration since stroke onset: 19.7 (0.4–94) months) before and after 3- to 4-week therapy. Both distribution-based (0.5 SD) and anchor-based (self-reported recovery score of SIS 3.0 as the external anchor) methods were used to estimate the MCID. The MCID for the EQ-Index was estimated to be 0.10 points using both anchor-based and distribution-based approaches. On the other hand, the MCID for the EQ-VAS was estimated to be 8.61 and 10.82 points, respectively [61].

18. **Physical Component Summary (PCS) score of the Short Form 36 (SF-36)**

The SF-36 (version 2 is described above) is a comprehensive patient-reported 36-item questionnaire that patients fill out to report on their health status, and it is widely used in clinical studies across a variety of health conditions. The survey encompasses eight domains that cover vitality, physical functioning, bodily pain, general health perceptions, emotional functioning, social functioning, as well as role limitations due to physical health problems and emotional problems. The SF-36 does not include any disease-specific questions. Possible scores range from 0 to 100, with higher scores indicating better health status. These scores can be Z-transformed and weighted to calculate Physical Component Summary (PCS) and Mental Component Summary (MCS) scores, which are norm-based with a mean of 50 and a standard deviation of 10. The PCS score is indicative of the person's physical health. The SF-36 has shown to have excellent psychometric properties and is widely used for people with stroke [62,63].

Fu et al., 2021, estimated the MCID of the PCS SF-36 score in 400 poststroke patients, utilizing data from a previous randomized controlled trial. Three methods were used to estimate the MCID: two anchor-based and one distribution-based method. A perceived health change (PHC) question was used as the anchor in the anchor-based method and the distribution-based method used 0.2 SD as the MCID. The estimated MCID was found to be 1.8 to 3.0 units, indicating the range of change in PCS scores that is considered to be clinically important for patients with stroke [64].

19. **6-Minute Walk Test (6MWT)**

The 6MWT is a commonly used clinical assessment tool used to evaluate functional exercise capacity in individuals with stroke. During the 6-minute walk test, the investigator will measure the patient's blood pressure, pulse, and oxygen level using a pulse oximeter before they start walking. The patient

should be given instructions to walk as far as possible for six minutes and to let the staff know if they experience chest pain or breathing difficulty. It is acceptable for the patient to slow down, rest, or stop during the test. The distance the patient can walk quickly on a flat, hard surface in six minutes is measured [65]. The Academy of Neurologic Physical Therapy strongly recommends the use of the 6-Minute Walk Test for individuals with stroke and other neurological conditions throughout the care continuum [66].

Fulk et al., 2018, utilized two external anchors, the Modified Rankin Scale (mRS) and Stroke Impact Scale (SIS), at 2 and 6 months post stroke to estimate the MCID. Patients two months post stroke received early locomotor training and a home exercise intervention for 12 to 16 weeks. With the mRS and the SIS as the anchors, the estimated MCIDs of the 6MWT were 71 meters (m) and 65 m, respectively, meaning that an increase in the distance covered of 65 to 71 meters during the 6MWT is considered to indicate a meaningful improvement in functional exercise capacity [67].

20. **Modified Ashworth Scale (MAS)**

The Modified Ashworth Scale (MAS) is a commonly used tool to measure spasticity in patients with neurological conditions such as stroke. The inter-rater reliability of MAS in stroke patients has been reported to be adequate and excellent [68,69]. The scale is an ordinal one with five points that range from 0, indicating no increase in muscle tone, to 4, indicating a rigid affected limb in either flexion or extension.

Chen et al. assessed the MCID of the MAS in 45 stroke patients who underwent rehabilitation, and the MAS was assessed at three different time points. A distribution-based method was used to estimate the MCID (0.5 SD and 0.8 SD). The MCID for the upper and lower extremity muscles based on effect sizes of 0.5 and 0.8 SD were found to be 0.48 and 0.76 for the upper extremity and 0.45 and 0.73 for the lower extremity, respectively [70].

21. **Functional Independence Measure (FIM) instrument**

The FIM instrument is a widely used functional performance measure developed specifically for the inpatient acute rehabilitation population [71]. It has been recommended by the Agency for Health Care Policy and Research Post-Stroke Rehabilitation panel as a measure of activities of daily living after stroke [72]. It is an 18-item instrument graded on a seven-point ordinal scale, with a maximum total score of 126. The seven-point ordinal scale indicates the burden of care associated with each aspect of function. The possible scores range from 1 (complete dependence) to 7 (independence). Various aspects of motor function are assessed, including eating, grooming, dressing, using the toilet, sphincter control, locomotion, and transfers, as well as several aspects of cognitive function including communication, memory, social, and problem solving. These aspects of function are commonly scored as a total score and also as motor and cognitive subscores. Subscores can also be tabulated for grouped aspects of function, such as self-care, sphincter control, transfers, locomotion, social, and communication [71]. The FIM has well-established reliability and validity as a generic instrument and in patients with stroke [73,74].

Beninato et al. evaluated the MCID of the FIM instrument in 113 poststroke patients (mean duration post stroke: 7.5 ± 5.8 days). Attending physicians rated assessments of clinical change on a 15-point Likert scale at discharge, and the MCID was defined at a cutoff score of 3. Receiver operating characteristic curves were used to identify FIM score changes associated with the MCID, and Bayesian analysis was utilized to determine the probability of individual patients achieving the MCID. Results showed that FIM score changes associated with the MCID were 22, 17, and 3 for the total FIM, motor FIM, and cognitive FIM, respectively [75].

22. **MCID for Safe and Simple Novel Acute Ischemic Stroke Therapies**

Cranston et al. explored the MCID for safe acute ischemic stroke treatment by administering an internet-based survey to 122 academic stroke neurologists. The survey's response framework was designed with a base of 1000 rather than the traditional base of 100. This was to prevent the respondents' answers from being skewed towards values in the 2% to 20% range. The survey was composed of five questions that were presented on a single web page. The first

four questions aimed to gather information about the respondents' characteristics, such as their appointment level, years of clinical practice, board certification, and the percentage of their practice devoted to stroke patients. The fifth question was focused on the MCID and presented a scenario with response options. The survey responses showed that expert-derived MCID is affected by anchoring and centrality bias, and the revised MCID value for safe acute ischemic stroke treatment is 1.1% to 1.5% [76].

23. **Montreal Cognitive Assessment (MoCA)**

Poststroke cognitive impairment can significantly affect a patient's capacity to carry out daily activities, potentially resulting in a reduced quality of life. It is important to identify and assess cognitive impairment in stroke patients in order to provide the appropriate interventions and support for improved outcomes. The MoCA (Montreal Cognitive Assessment) is a commonly used cognitive screening tool to assess cognitive impairment. It measures various domains including visuospatial abilities, naming, attention, language, abstraction, delayed recall, and orientation. Total scores range from 0 to 30, with a score of 26 or above considered normal cognitive function. One point is added to the total score for participants who have received less than 12 years of education, as lower education levels can impact performance on the test [77–79].

Wu et al. estimated the MCID of the MoCA in 65 stroke patients (mean duration after stroke onset: 20 23 ± 13 48 months) before and after 4 to 5 weeks of therapy. Both anchor (using patient-perceived recovery score of the SIS 3.0 scale as external anchor) and distribution-based methods (0.5 SD) were used. The MCID for the MoCA was estimated at 1.22 points using the anchor-based method and 2.15 points using the distribution-based method [80].

24. **Stroke-Specific Quality of Life (SS-QOL)**

The Stroke-Specific Quality Of Life scale (SS-QOL) is a self-reported questionnaire intended to provide an assessment of health-related quality of life (HRQoL) specific to patients with stroke[1]. It assesses health-related quality of life specific to stroke survivors. The SS-QOL was developed for use in patients with stroke. It has 49 items and assesses eight domains: energy, family roles, language, mobility, mood, personality, self-care, and social roles. The Stroke-Specific Quality of Life Scale (SS-QOL) has a score range of 49 to 245 points, with higher scores indicating a better HRQoL [81].

Lin et al. assessed the MCID for the SS-QOL in 84 stroke patients at a mean poststroke duration of 18.1 months. Both anchor (SIS as anchor) and distribution (0.5 SD)-based methods were used to estimate the MCID. The MCID for the three subscales, viz. mobility, self-care, and upper extremity (UE) function, were 1.5 to 2.4, 1.2 to 1.9, and 1.2 to 1.8, respectively [82].

References for Supplementary Material

1. Bland MD, Sturmoski A, Whitson M, Connor LT, Fucetola R, Huskey T, et al. Prediction of discharge walking ability from initial assessment in a stroke inpatient rehabilitation facility population. Arch Phys Med Rehabil. 2012 Aug;93(8):1441–7.

2. Blum L, Korner-Bitensky N. Usefulness of the Berg Balance Scale in stroke rehabilitation: a systematic review. Phys Ther. 2008 May;88(5):559–66.

3. Berg Balance Scale - an overview | ScienceDirect Topics [Internet]. [cited 2023 Apr 21]. Available from: https://www.sciencedirect.com/topics/medicine-and-dentistry/berg-balance-scale

4. Tamura S, Miyata K, Kobayashi S, Takeda R, Iwamoto H. The minimal clinically important difference in Berg Balance Scale scores among patients with early subacute stroke: a multicenter, retrospective, observational study. Top Stroke Rehabil. 2021 Jun 25;1–7.

5. Song MJ, Lee JH, Shin WS. Minimal Clinically Important Difference of Berg Balance Scale scores in people with acute stroke. Physical Therapy Rehabilitation Science. 2018 Sep 30;7(3):102–8.

6. Di Carlo S, Bravini E, Vercelli S, Massazza G, Ferriero G. The Mini-BESTest: a review of psychometric properties. Int J Rehabil Res. 2016 Jun;39(2):97–105.

7. Tsang CSL, Liao LR, Chung RCK, Pang MYC. Psychometric properties of the Mini-Balance Evaluation Systems Test (Mini-BESTest) in community-dwelling individuals with chronic stroke. Phys Ther. 2013 Aug;93(8):1102–15.

8. Franchignoni F, Horak F, Godi M, Nardone A, Giordano A. Using psychometric techniques to improve the Balance Evaluation Systems Test: the mini-BESTest. J Rehabil Med. 2010 Apr;42(4):323–31.
9. Beauchamp MK, Niebuhr R, Roche P, Kirkwood R, Sibley KM. A prospective study to establish the minimal clinically important difference of the Mini-BESTest in individuals with stroke. Clin Rehabil. 2021 Jun 15;2692155211025131.
10. Thompson M, Medley A, Teran S. Validity of the Sitting Balance Scale in older adults who are non-ambulatory or have limited functional mobility. Clin Rehabil. 2013 Feb 1;27(2):166–73.
11. Sandin KJ, Smith BS. The measure of balance in sitting in stroke rehabilitation prognosis. Stroke. 1990 Jan;21(1):82–6.
12. Gorman SL, Radtka S, Melnick ME, Abrams GM, Byl NN. Development and Validation of the Function In Sitting Test in Adults With Acute Stroke. Journal of Neurologic Physical Therapy. 2010 Sep;34(3):150.
13. Alzyoud J, Medley A, Thompson M, Csiza L. Responsiveness, minimal detectable change, and minimal clinically important difference of the sitting balance scale and function in sitting test in people with stroke. Physiother Theory Pract. 2020 May 13;1–10.
14. Whitney SL, Wrisley DM, Marchetti GF, Gee MA, Redfern MS, Furman JM. Clinical measurement of sit-to-stand performance in people with balance disorders: validity of data for the Five-Times-Sit-to-Stand Test. Phys Ther. 2005 Oct;85(10):1034–45.
15. Jones SE, Kon SSC, Canavan JL, Patel MS, Clark AL, Nolan CM, et al. The five-repetition sit-to-stand test as a functional outcome measure in COPD. Thorax. 2013 Nov;68(11):1015–20.
16. Bernabeu-Mora R, Giménez-Giménez LM, Montilla-Herrador J, García-Guillamón G, García-Vidal JA, Medina-Mirapeix F. Determinants of each domain of the Short Physical Performance Battery in COPD. Int J Chron Obstruct Pulmon Dis. 2017;12:2539–44.
17. Agustín RMS, Crisostomo MJ, Sánchez-Martínez MP, Medina-Mirapeix F. Responsiveness and Minimal Clinically Important Difference of the Five Times Sit-to-Stand Test in Patients with Stroke. Int J Environ Res Public Health. 2021 Feb 26;18(5):2314.
18. Cooper A, Alghamdi GA, Alghamdi MA, Altowaijri A, Richardson S. The Relationship of Lower Limb Muscle Strength and Knee Joint Hyperextension during the Stance Phase of Gait in Hemiparetic Stroke Patients. Physiotherapy Research International. 2012;17(3):150–6.
19. Beyaert C, Vasa R, Frykberg GE. Gait post-stroke: Pathophysiology and rehabilitation strategies. Neurophysiol Clin. 2015 Nov;45(4–5):335–55.
20. Guzik A, Drużbicki M, Wolan-Nieroda A, Turolla A, Kiper P. Estimating Minimal Clinically Important Differences for Knee Range of Motion after Stroke. J Clin Med. 2020 Oct 15;9(10):E3305.
21. Wellmon R, Degano A, Rubertone JA, Campbell S, Russo KA. Interrater and intrarater reliability and minimal detectable change of the Wisconsin Gait Scale when used to examine videotaped gait in individuals post-stroke. Arch Physiother. 2015 Oct 5;5:11.
22. Guzik A, Drużbicki M, Wolan-Nieroda A, Przysada G, Kwolek A. The Wisconsin gait scale - The minimal clinically important difference. Gait Posture. 2019 Feb;68:453–7.
23. O�Neil KH, Purdy M, Falk J, Gallo L. The Dysphagia Outcome and Severity Scale. Dysphagia. 1999;14(3):139–45.
24. Everton LF, Benfield JK, Hedstrom A, Wilkinson G, Michou E, England TJ, et al. Psychometric assessment and validation of the dysphagia severity rating scale in stroke patients. Sci Rep. 2020 Apr 29;10(1):7268.
25. Kopp B, Kunkel A, Flor H, Platz T, Rose U, Mauritz KH, et al. The Arm Motor Ability Test: reliability, validity, and sensitivity to change of an instrument for assessing disabilities in activities of daily living. Arch Phys Med Rehabil. 1997 Jun;78(6):615–20.
26. Fulk G, Martin R, Page SJ. Clinically Important Difference of the Arm Motor Ability Test in Stroke Survivors. Neurorehabil Neural Repair. 2017 Mar;31(3):272–9.
27. Daley K, Mayo N, Wood-Dauphinée S. Reliability of Scores on the Stroke Rehabilitation Assessment of Movement (STREAM) Measure. Physical Therapy. 1999 Jan 1;79(1):8–23.
28. Hsieh YW, Wang CH, Sheu CF, Hsueh IP, Hsieh CL. Estimating the minimal clinically important difference of the Stroke Rehabilitation Assessment of Movement measure. Neurorehabil Neural Repair. 2008 Dec;22(6):723–7.
29. Duncan PW, Sullivan KJ, Behrman AL, Azen SP, Wu SS, Nadeau SE, et al. Body-weight-supported treadmill rehabilitation after stroke. N Engl J Med. 2011 May 26;364(21):2026–36.

30. Lai SM, Perera S, Duncan PW, Bode R. Physical and social functioning after stroke: comparison of the Stroke Impact Scale and Short Form-36. Stroke. 2003 Feb;34(2):488–93.
31. Fulk GD, Ludwig M, Dunning K, Golden S, Boyne P, West T. How much change in the stroke impact scale-16 is important to people who have experienced a stroke? Top Stroke Rehabil. 2010 Dec;17(6):477–83.
32. Nouri F, Lincoln N. An extended activities of daily living scale for stroke patients. Clin Rehabil. 1987 Nov 1;1(4):301–5.
33. Chong DK. Measurement of instrumental activities of daily living in stroke. Stroke. 1995 Jun;26(6):1119–22.
34. Salter KL, Teasell RW, Foley NC, Jutai JW. Outcome assessment in randomized controlled trials of stroke rehabilitation. Am J Phys Med Rehabil. 2007 Dec;86(12):1007–12.
35. Wu C yi, Chuang L ling, Lin K chung, Lee S da, Hong W hsien. Responsiveness, minimal detectable change, and minimal clinically important difference of the Nottingham Extended Activities of Daily Living Scale in patients with improved performance after stroke rehabilitation. Arch Phys Med Rehabil. 2011 Aug;92(8):1281–7.
36. Urbin MA, Waddell KJ, Lang CE. Acceleration metrics are responsive to change in upper extremity function of stroke survivors. Arch Phys Med Rehabil. 2015 May;96(5):854–61.
37. Accelerometer - an overview | ScienceDirect Topics [Internet]. [cited 2023 Apr 23]. Available from: https://www.sciencedirect.com/topics/materials-science/accelerometer
38. Chen HL, Lin KC, Hsieh YW, Wu CY, Liing RJ, Chen CL. A study of predictive validity, responsiveness, and minimal clinically important difference of arm accelerometer in real-world activity of patients with chronic stroke. Clin Rehabil. 2018 Jan;32(1):75–83.
39. de Morton NA, Davidson M, Keating JL. The de Morton Mobility Index (DEMMI): An essential health index for an ageing world. Health and Quality of Life Outcomes. 2008 Aug 19;6(1):63.
40. New PW, Scroggie GD, Williams CM. The validity, reliability, responsiveness and minimal clinically important difference of the de Morton mobility index in rehabilitation. Disabil Rehabil. 2017 May;39(10):1039–43.
41. Cimolin V, Galli M, Vimercati SL, Albertini G. Use of the Gait Deviation Index for the assessment of gastrocnemius fascia lengthening in children with Cerebral Palsy. Res Dev Disabil. 2011;32(1):377–81.
42. Correa KP, Devetak GF, Martello SK, de Almeida JC, Pauleto AC, Manffra EF. Reliability and Minimum Detectable Change of the Gait Deviation Index (GDI) in post-stroke patients. Gait Posture. 2017 Mar;53:29–34.
43. Bertrand AM, Mercier C, Bourbonnais D, Desrosiers J, Gravel D. Reliability of maximal static strength measurements of the arms in subjects with hemiparesis. Clin Rehabil. 2007 Mar;21(3):248–57.
44. Schmidt RT, Toews JV. Grip strength as measured by the Jamar dynamometer. Arch Phys Med Rehabil. 1970 Jun;51(6):321–7.
45. Andrews AW, Thomas MW, Bohannon RW. Normative values for isometric muscle force measurements obtained with hand-held dynamometers. Phys Ther. 1996 Mar;76(3):248–59.
46. Wagner JM, Lang CE, Sahrmann SA, Edwards DF, Dromerick AW. Sensorimotor impairments and reaching performance in subjects with poststroke hemiparesis during the first few months of recovery. Phys Ther. 2007 Jun;87(6):751–65.
47. Platz T, Pinkowski C, van Wijck F, Kim IH, di Bella P, Johnson G. Reliability and validity of arm function assessment with standardized guidelines for the Fugl-Meyer Test, Action Research Arm Test and Box and Block Test: a multicentre study. Clin Rehabil. 2005 Jun;19(4):404–11.
48. Van der Lee JH, De Groot V, Beckerman H, Wagenaar RC, Lankhorst GJ, Bouter LM. The intra- and interrater reliability of the action research arm test: a practical test of upper extremity function in patients with stroke. Arch Phys Med Rehabil. 2001 Jan;82(1):14–9.
49. Wolf SL, Catlin PA, Ellis M, Archer AL, Morgan B, Piacentino A. Assessing Wolf motor function test as outcome measure for research in patients after stroke. Stroke. 2001 Jul;32(7):1635–9.
50. Morris DM, Uswatte G, Crago JE, Cook EW, Taub E. The reliability of the wolf motor function test for assessing upper extremity function after stroke. Arch Phys Med Rehabil. 2001 Jun;82(6):750–5.
51. van der Lee JH, Beckerman H, Knol DL, de Vet HCW, Bouter LM. Clinimetric properties of the motor activity log for the assessment of arm use in hemiparetic patients. Stroke. 2004 Jun;35(6):1410–4.
52. Uswatte G, Taub E, Morris D, Vignolo M, McCulloch K. Reliability and validity of the upper-extremity Motor Activity Log-14 for measuring real-world arm use. Stroke. 2005 Nov;36(11):2493–6.

53. Lang CE, Wagner JM, Edwards DF, Dromerick AW. Upper extremity use in people with hemiparesis in the first few weeks after stroke. J Neurol Phys Ther. 2007 Jun;31(2):56–63.
54. Uswatte G, Miltner WH, Foo B, Varma M, Moran S, Taub E. Objective measurement of functional upper-extremity movement using accelerometer recordings transformed with a threshold filter. Stroke. 2000 Mar;31(3):662–7.
55. Lang CE, Edwards DF, Birkenmeier RL, Dromerick AW. Estimating minimal clinically important differences of upper-extremity measures early after stroke. Arch Phys Med Rehabil. 2008 Sep;89(9):1693–700.
56. Penta M, Thonnard JL, Tesio L. ABILHAND: a Rasch-built measure of manual ability. Arch Phys Med Rehabil. 1998 Sep;79(9):1038–42.
57. Wang T ni, Lin K chung, Wu C yi, Chung C ying, Pei Y cheng, Teng Y kuei. Validity, responsiveness, and clinically important difference of the ABILHAND questionnaire in patients with stroke. Arch Phys Med Rehabil. 2011 Jul;92(7):1086–91.
58. EuroQol Group. EuroQol--a new facility for the measurement of health-related quality of life. Health Policy. 1990 Dec;16(3):199–208.
59. User⊛ manual for the SF36v2 Health Survey – ScienceOpen [Internet]. [cited 2023 Feb 7]. Available from: https://www.scienceopen.com/document?vid=0a250605-f5f8-489a-a73c-329de570f424
60. Kim SK, Kim SH, Jo MW, Lee S il. Estimation of minimally important differences in the EQ-5D and SF-6D indices and their utility in stroke. Health Qual Life Outcomes. 2015 Mar 9;13:32.
61. Chen P, Lin KC, Liing RJ, Wu CY, Chen CL, Chang KC. Validity, responsiveness, and minimal clinically important difference of EQ-5D-5L in stroke patients undergoing rehabilitation. Qual Life Res. 2016 Jun;25(6):1585–96.
62. Anderson C, Laubscher S, Burns R. Validation of the Short Form 36 (SF-36) health survey questionnaire among stroke patients. Stroke. 1996 Oct;27(10):1812–6.
63. Ware J, Snoww K, MA K, BG G. SF36 Health Survey: Manual and Interpretation Guide. Lincoln, RI: Quality Metric, Inc, 1993. 1993 Jan 1;30.
64. Fu V, Weatherall M, McNaughton H. Estimating the minimal clinically important difference for the Physical Component Summary of the Short Form 36 for patients with stroke. J Int Med Res. 2021 Dec;49(12):3000605211067902.
65. Duncan PW, Wallace D, Lai SM, Johnson D, Embretson S, Laster LJ. The Stroke Impact Scale Version 2.0. Stroke. 1999 Oct;30(10):2131–40.
66. Moore JL, Potter K, Blankshain K, Kaplan SL, O'Dwyer LC, Sullivan JE. A Core Set of Outcome Measures for Adults With Neurologic Conditions Undergoing Rehabilitation: A CLINICAL PRACTICE GUIDELINE. J Neurol Phys Ther. 2018 Jul;42(3):174–220.
67. Fulk GD, He Y. Minimal Clinically Important Difference of the 6-Minute Walk Test in People With Stroke. J Neurol Phys Ther. 2018 Oct;42(4):235–40.
68. Blackburn M, van Vliet P, Mockett SP. Reliability of measurements obtained with the modified Ashworth scale in the lower extremities of people with stroke. Phys Ther. 2002 Jan;82(1):25–34.
69. Bohannon RW, Smith MB. Interrater reliability of a modified Ashworth scale of muscle spasticity. Phys Ther. 1987 Feb;67(2):206–7.
70. Chen CL, Chen CY, Chen HC, Wu CY, Lin KC, Hsieh YW, et al. Responsiveness and minimal clinically important difference of Modified Ashworth Scale in patients with stroke. Eur J Phys Rehabil Med. 2019 Dec;55(6):754–60.
71. Granger CV, Hamilton BB, Keith RA, Zielezny M, Sherwin FS. Advances in functional assessment for medical rehabilitation. Topics in Geriatric Rehabilitation. 1986 Apr;1(3):59.
72. Gresham GE, Duncan PW, Stason WB, Adams HP, Adelman AM, Alexander DN, et al. Post-Stroke Rehabilitation. Clinical Practice Guideline Number 16; Post-Stroke Rehabilitation: Assessment, Referral, and Patient Management. Quick Reference Guide for Clinicians Number 16; Recovering After a Stroke, Consumer Version. Patient and Family Guide. [Internet]. Agency for Health Care Policy and Research, Rockville, MD. Center for Research Dissemination and Liaison.; 1995 [cited 2023 Apr 24]. Report No.: PB95226890. Available from: https://ntrl.ntis.gov/NTRL/dashboard/searchResults/titleDetail/PB95226890.xhtml
73. Ottenbacher KJ, Hsu Y, Granger CV, Fiedler RC. The reliability of the functional independence measure: a quantitative review. Arch Phys Med Rehabil. 1996 Dec;77(12):1226–32.
74. Dodds TA, Martin DP, Stolov WC, Deyo RA. A validation of the functional independence measurement and its performance among rehabilitation inpatients. Arch Phys Med Rehabil. 1993 May;74(5):531–6.

75. Beninato M, Gill-Body KM, Salles S, Stark PC, Black-Schaffer RM, Stein J. Determination of the minimal clinically important difference in the FIM instrument in patients with stroke. Arch Phys Med Rehabil. 2006 Jan;87(1):32–9.
76. Cranston JS, Kaplan BD, Saver JL. Minimal Clinically Important Difference for Safe and Simple Novel Acute Ischemic Stroke Therapies. Stroke. 2017 Nov;48(11):2946–51.
77. Nasreddine ZS, Phillips NA, BÃ©dirian V, Charbonneau S, Whitehead V, Collin I, et al. The Montreal Cognitive Assessment, MoCA: A Brief Screening Tool For Mild Cognitive Impairment: MOCA: A BRIEF SCREENING TOOL FOR MCI. Journal of the American Geriatrics Society. 2005 Apr;53(4):695–9.
78. MoCA Cognition [Internet]. [cited 2023 Feb 13]. MoCA Cognition. Available from: https://mocacognition.com/
79. Park JH, Kim BJ, Bae HJ, Lee J, Lee J, Han MK, et al. Impact of post-stroke cognitive impairment with no dementia on health-related quality of life. J Stroke. 2013 Jan;15(1):49–56.
80. Wu CY, Hung SJ, Lin KC, Chen KH, Chen P, Tsay PK. Responsiveness, Minimal Clinically Important Difference, and Validity of the MoCA in Stroke Rehabilitation. Occup Ther Int. 2019;2019:2517658.
81. Pedersen SG, Friborg O, Heiberg GA, Arntzen C, Stabel HH, Thrane G, et al. Stroke-Specific Quality of Life one-year post-stroke in two Scandinavian country-regions with different organisation of rehabilitation services: a prospective study. Disability and Rehabilitation. 2021 Dec 18;43(26):3810–20.
82. Lin K chung, Fu T, Wu C yi, Hsieh C ju. Assessing the stroke-specific quality of life for outcome measurement in stroke rehabilitation: minimal detectable change and clinically important difference. Health Qual Life Outcomes. 2011 Jan 19;9:5.
